# Integrated Module and Gene-Specific Regulatory Inference Implicates Upstream Signaling Networks

**DOI:** 10.1371/journal.pcbi.1003252

**Published:** 2013-10-17

**Authors:** Sushmita Roy, Stephen Lagree, Zhonggang Hou, James A. Thomson, Ron Stewart, Audrey P. Gasch

**Affiliations:** 1 Department of Biostatistics and Medical Informatics, University of Wisconsin-Madison, Madison, Wisconsin, United States of America; 2 Wisconsin Institute for Discovery, Madison, Wisconsin, United States of America; 3 Department of Computer Science, University of Wisconsin-Madison, Madison, Wisconsin, United States of America; 4 Morgridge Institute for Research, Madison, Wisconsin, United States of America; 5 Department of Cell and Regenerative Biology, University of Wisconsin-Madison, Madison, Wisconsin, United States of America; 6 Department of Molecular, Cellular, and Developmental Biology, University of California Santa Barbara, Santa Barbara, California, United States of America; 7 Department of Genetics, University of Wisconsin-Madison, Madison, Wisconsin, United States of America; National Center for Biotechnology Information (NCBI), United States of America

## Abstract

Regulatory networks that control gene expression are important in diverse biological contexts including stress response and development. Each gene's regulatory program is determined by module-level regulation (e.g. co-regulation via the same signaling system), as well as gene-specific determinants that can fine-tune expression. We present a novel approach, *Modular regulatory network learning with per gene information* (MERLIN), that infers regulatory programs for individual genes while probabilistically constraining these programs to reveal module-level organization of regulatory networks. Using edge-, regulator- and module-based comparisons of simulated networks of known ground truth, we find MERLIN reconstructs regulatory programs of individual genes as well or better than existing approaches of network reconstruction, while additionally identifying modular organization of the regulatory networks. We use MERLIN to dissect global transcriptional behavior in two biological contexts: yeast stress response and human embryonic stem cell differentiation. Regulatory modules inferred by MERLIN capture co-regulatory relationships between signaling proteins and downstream transcription factors thereby revealing the upstream signaling systems controlling transcriptional responses. The inferred networks are enriched for regulators with genetic or physical interactions, supporting the inference, and identify modules of functionally related genes bound by the same transcriptional regulators. Our method combines the strengths of per-gene and per-module methods to reveal new insights into transcriptional regulation in stress and development.

## Author Summary

The state of a cell is largely determined by the genes the cell expresses. Transcriptional control of gene expression is exerted by transcription factor proteins that bind to regulatory regions of genes and affect their expression. Transcriptional programs have a modular organization enabling multiple genes to be coordinately regulated, and at the same time are fine-tuned for each gene through interactions of transcription factors with a gene's regulatory region. Transcription factors are themselves controlled by upstream signaling proteins, that in turn can be transcriptionally controlled. This complex process of gene expression control is described by a regulatory network that captures who regulates whom. A key challenge in systems biology is to reconstruct regulatory networks that capture precise gene-specific regulatory information, as well as the modular organization of transcriptional programs. We developed a novel regulatory network inference approach, MERLIN, *Modular regulatory network learning with per gene information*. When applied to examine transcriptional responses in two distinct processes, stress response and cellular differentiation, MERLIN accurately reconstructed regulatory programs of individual genes while revealing regulatory module organization and predicted upstream signaling proteins for regulatory modules. MERLIN is applicable to different environmental, developmental and disease contexts to dissect regulatory programs and ultimately build network-based predictive models of cellular states.

## Introduction

Regulatory networks that connect regulators (signaling proteins and transcription factors) to target genes are core information processing components in cells and control *what* genes must be expressed *when* [1]–[3]. Eukaryotic regulatory networks have several organizational properties: (1) regulatory networks are modular, enabling multiple genes to be simultaneously regulated through the same regulatory mechanisms [4], [5], (2) individual genes are often regulated by multiple transcription factors that combinatorially bind to promoters of genes [6]–[9]. Activation of upstream signaling proteins and their downstream transcription factors alters global gene expression in dynamic ways and often, upstream regulators are themselves regulated, via feedback and feed-forward loops [2], [10]. These dynamic patterns can be readily quantified through advances in regulatory genomics, enabling us to describe cellular states by signature patterns of expression and chromatin modifications. Computational reconstruction of regulatory networks provide a powerful approach to dissect these states relying on the premise that the expression patterns of genes encoding upstream regulators are predictive of the expression of other target genes of that signaling system [11]–[16]. A major challenge that remains is to combine these regulatory network properties of individual genes and sets of genes in a module to build predictive models of system state.

**Figure 1:**
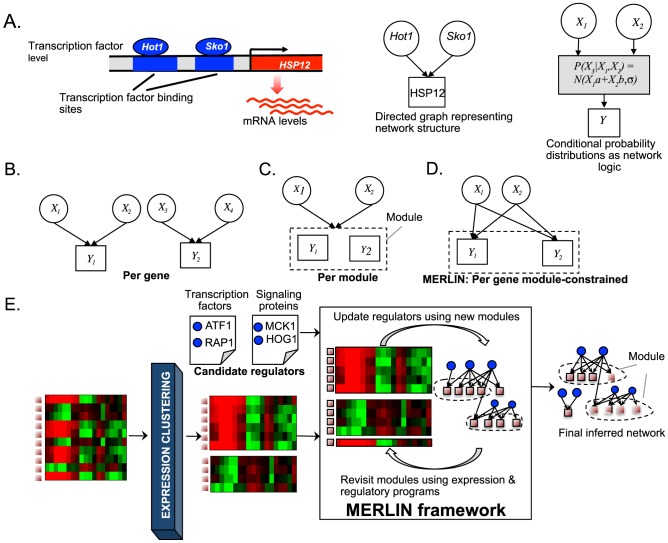
Per-gene and per-module regulatory network inference approaches. **A.** Modeling transcriptional regulatory networks as a probabilistic graphical model. Shown is a cartoon of a gene promoter HSP12, with two regulators that bind to its promoter to regulate its level. Regulatory networks are represented using a directed graph specifying who regulates whom, with arrows from regulators to target genes. The network logic of how the regulator levels predicts the target gene expression level is modeled through conditional probability distributions in a probabilistic graphical model. **B.** Per-gene regulatory network learning. Regulators for each gene are inferred independently. **C.** Per-module network inference. Regulators are inferred for each module. All genes in the same module have the same parameters. **D.** Per-gene module constrained network learning used in MERLIN. Gene-specific regulatory programs are inferred while imposing module constraints to enable genes in the same module to share regulators. **E.** MERLIN learning framework for inferring regulatory module networks. The algorithm starts with an initial set of expression clusters and candidate regulators and iterates between learning regulatory programs for each gene, and revisiting the module membership. The final inferred network is the output of MERLIN comprising the per-gene regulatory programs and the module membership of each gene.

Computational methods for network reconstruction can be broadly classified into two groups: (1) per-gene methods (**Figure 1B**), which infer a regulatory network one gene at a time [17]–[21], and (2) per-module methods [14], [22], [23] (**Figure 1C**), which infer a regulatory network by grouping similarly expressed genes into modules and inferring a single regulatory program for the module. While the per-gene methods can infer precise regulatory logic of every gene, considering each gene separately ignores the modular organization of networks. On the other hand, per-module approaches learn concise and modular structures, but they simplify the regulatory network by requiring all genes in the module to have the same regulatory program. This simplification comes at the cost of important regulatory information at individual genes, such as variations in transcription factor interactions due to gene-specific promoter architecture. Thus, while per-module approaches succeed in identifying regulators that affect larger module-level behavior, they cannot identify the regulators that are important from an individual genes perspective because they do not incorporate gene-specific parameters.

We propose a novel regulatory network reconstruction approach, *Modular regulatory network learning with per gene information*, MERLIN, that combines the strengths of per-gene and per-module network inference methods (**Figure 1D**). Specifically, our approach learns separate regulatory programs for each gene, but constrains the network using a probabilistic graphical model such that genes in the same module have similar, but not identical, regulatory programs. Furthermore the algorithm learns both the network structure and network parameters that can be used to predict expression in a test condition.

Comparison of our approach to state of the art per-gene and per-module methods clearly identified the strengths of our approach in accurately recovering both edge and module-based regulatory information. We applied our method to published transcriptome measurements of yeast stress responses [24] and a new human embryonic stem cell differentiation dataset. In both processes, MERLIN inferred transcription factors and signaling proteins that work in concert to regulate the same module, allowing us to predict the upstream signaling networks that function together in the cell. We identify regulatory networks recapitulating the combinatorial transcriptional control of amino acid metabolism genes [7], [24], and additionally implicate the HOG1 MAP kinase to be the upstream regulator of numerous modules associated with osmotic and cell wall stress. In humans, we predict regulators from major signaling pathways including Notch and Hedgehog pathways for modules associated with the maintenance of pluripotency and with the onset of cellular differentiation.

## Results

### MERLIN: An approach to capture per-gene and per-module regulatory information

A mathematical model of a regulatory network has two components: the *structure* specifies the regulators of a target gene, and the *logic*, encoded in mathematical functions, describes the sign and magnitude of individual and combinations of regulators that specify the expression of that gene. Several different mathematical functions could relate the expression of the upstream regulators to the mRNA level of a target, e.g. boolean functions, differential equations, probabilistic functions. The MERLIN approach is based on a probabilistic graphical model representation of a regulatory network (**Figure 1A**) [17], [21], [25], [26]. Within our probabilistic graphical model, both genes and their regulators (which can be targets as well) are represented as random variables whose associated probability distributions represent the range of values a gene can take in different microarray or RNA-seq experiments. In probabilistic graphical models, the mathematical functions relating the level of a regulator to the level of a gene is a conditional probability distribution, specifying the probability of a target gene to take a specific expression value given the expression values of its regulators. We use a conditional Gaussian model for the conditional distribution, with mean of the Gaussian derived as a linear function of the expression levels of the regulators (See **Materials and Methods**).

We assume that we have measured expression levels of both gene targets and encoded regulators under multiple conditions, and regulators and target genes co-vary under different conditions. To reconstruct the regulatory network from given gene expression data we need to infer both the structure as well as parameters of the mathematical functions. Our network inference approach, MERLIN, combines the two popular strategies of expression-based network inference approaches described above: per-gene [18], [19], and per-module [14] approaches. MERLIN learns the gene-specific regulatory programs while imposing a module constraint as a probabilistic prior. Instead of selecting regulators independently for each gene 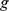, the prior enables us to take into account regulators that are predicted to regulate other genes co-expressed with 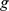 in the same module. In this way we impose a “soft” module constraint so that two genes in a module are favored to have similar but not necessarily identical regulators as in a per-module approach.

The MERLIN learning algorithm begins with a set of modules, which are typically defined by an expression-based clustering step, and a set of candidate regulators of gene expression (e.g. all transcription factors, kinases, phosphatases annotated in an organism). It then iterates over two steps (**Figure 1E**): (a) a regulator identification step, and (b) a module inference step. In the regulator identification, the modules are kept fixed and the regulator sets of each gene are identified by adding new regulators that reduce the prediction error of a gene 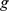
's expression value from the expression values of the regulators, while using a probabilistic “module” prior on the graph that favors regulators regulating other genes in 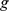
's module, (**Materials and Methods**). The prior enables us to favor graph structures that are more modular. In the module inference step, genes are grouped into modules using co-expression and co-regulation based on the inferred set of regulators for a pair of genes. Co-expression is measured by the Pearson's correlation between two gene expression profiles. Co-regulation is measured by the similarity of inferred regulators for each gene (**Materials and Methods**). The algorithm repeats these two phases of the algorithm until convergence. In addition to the module prior, we also use a model complexity prior that penalizes excessive parameters in the model (for example, due to a large number of regulators). Such a complexity prior avoids over-fitting the model to the data. Both the structure complexity prior and the module prior are controlled by user-defined parameters, and can be flexibly adjusted to control how strongly we want to impose each prior.

### MERLIN accurately infers per-gene regulatory programs as well as module-level organization on both simulated ground truth and real expression data

We compared the quality of networks inferred by MERLIN to those inferred from three other algorithms using several criteria defined below. The algorithms include a linear regression per-gene network inference method (LINEARREGR), GENIE3 a state-of-the-art per-gene network inference algorithm [19], and Module networks (MODNET) from Segal et al. [14]. The LINEARREGR approach that we used is a special case of MERLIN where we set the module prior to zero; this served as the baseline to study the gain in performance by adding the “module” constraint in our MERLIN approach. We also considered a Bayesian network as a baseline (**Figure S1**), but the performance was much worse than any of the methods above.

We used both simulated gene expression data where the ground truth of the networks generating the data were known, as well as real gene expression data where the ground truth networks are not known. Simulated data was generated for networks of different number of genes, *n* = 100, 200, 300, 400, 500, 1000 genes using GeneNetWeaver (GNW) [27]. This simulator takes networks as inputs and uses stochastic differential equations to generate simulated expression data. The simulated data had 100, 200, 300, 400, 500 and 1,000 measurements generated by perturbing one node and propagating the system to steady state. The simulated networks were generated so that they were modular, that is, genes in the same module tended to share more regulators than genes in different modules (**Materials and Methods**).

**Figure 2:**
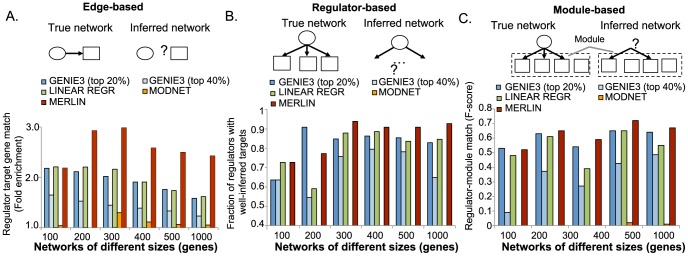
Comparison of MERLIN against per-gene and per-module network inference algorithms using simulated data. **A.** Comparison based on fold enrichment of true edges in the inferred network. The cartoon illustrates that this metric compares each edge in isolation. The fold enrichment is positive, and higher it is the better the inferred network in terms of the true edges recovered, and the false edges not inferred. **B.** Fraction of regulators whose targets in the true network are significantly overlapping with its targets in the inferred network (higher is better). The cartoons shows that this metric compares a set of genes, namely, the targets of a regulator. Each network had different numbers of total regulators, NET100: 11, NET200: 22, NET300: 33, NET400: 44, NET500: 55, NET1000: 111, which we used to obtain a fraction of regulators in each network. **C.** Overlap as measured by F-score between regulator-module relationships in the true network and regulator-module relationships from the inferred networks. The cartoon shows this metric compares networks based on the regulators associated with known modules. F-score ranges from 0 to 1, and the closer it is to 1 the better the performance.

We defined three criteria to compare the inferred networks: (a) **Edge-based comparison** used fold enrichment and the area under the precision-recall curve (AUPR) [28], to assess edge overlap between the simulated ground truth network and inferred networks (**Figure 2A**, **Figure S1A**, **Materials and Methods**), (b) **Regulator-based comparison** measured the number of regulators whose targets significantly matched between the true and inferred networks (FDR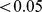, **Figure 2B**), (c) **Module-based comparison** measured how many regulators associated with modules in the ground truth networks matched regulators associated with these modules in the inferred networks (**Figure 2C**). The AUPR edge-based comparison requires a ranking of edges and does not require us to specify a particular cutoff. The edge-based fold enrichment, regulator- and module-based measures require use to define a network. Because GENIE3 does not provide a discrete network but rather a ranking of all edges, and since the edge ranking does not translate into edge confidence, we considered networks with the top 20% or the top 40% edges from the GENIE3 output.

On simulated data MERLIN outperformed LINEARREGR and MODNET using edge-, regulator- and module-based metrics (**Figure 2A–C**), suggesting that adding a module prior is beneficial for inferring better networks. MERLIN was significantly better than GENIE3 using fold enrichment (t-test 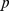-value <0.003), and both approaches were at par using AUPR (**Figure 2A**, t-test 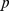-value = 0.5), suggesting an overall improved performance than GENIE3. On both regulator- and module-based metrics GENIE3's performance depended greatly on the threshold used to define a network; in no case was it better than MERLIN, but significantly worse on the module-regulator relations than MERLIN (t-test 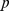-value <0.04). We were surprised to see that MODNET did not perform well on the simulated network datasets. This is likely due to the extremely sparse networks MODNET infers. We observe a more comparable performance, although still low, when considering the yeast regulatory networks.

**Figure 3:**
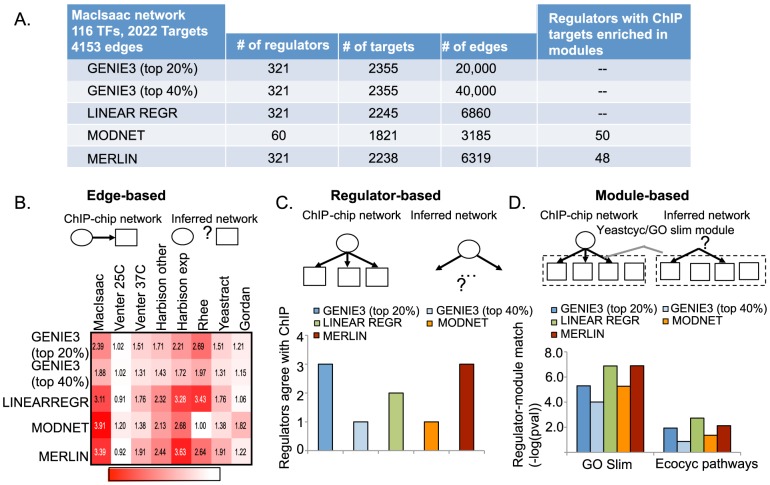
Comparison of MERLIN against per-gene and per-module network inference algorithms using a large compendia of gene expression data in yeast stress response. **A.** Shown are the various network statistics of the inferred networks. The last column in the table shows the number of regulators with targets significantly overlapping between ChIP targets and modules. **B.** Comparison based on fold enrichment of physical regulatory edges measured using various experimental methods such as ChIP-chip, in the inferred networks. The more red, the higher the fold enrichment and the better the inferred network is able to capture these physical interactions. The columns correspond to the different experimentally derived networks: ChIP-chip (Harbison, Venters), ChIP-exo (Rhee), and protein binding microarrays followed by manual curation (Gordan). **C.** Number of regulators whose targets in the true network are significantly overlapping with its targets in the inferred network. **D.** Overlap as measured by −log(pval) between regulator-module relationships in the ChIP-chip network from MacIsaac et al. A module here corresponds to either a Yeastcyc pathway or set of genes annotated with a particular GO slim process term.

We next compared the network inference algorithms using a well-studied yeast dataset from Gasch et al [24] comprising 2,355 genes and 466 candidate regulators catalogued in Segal et al. [14], where regulators included both transcription factors as well as signaling proteins such as kinases or phosphatases (**Figure 3A**). Because the ground truth network is not available, we assessed the quality of the inferred networks based on their overlap with other reconstructions of yeast transcriptional regulatory networks using ChIP-chip [6], [9], ChIP-exo [29], evolutionary conservation [30], and curated TF motifs from protein binding microarrays [31]. These networks include edges with some experimental evidence to suggest the presence of a regulatory edge: (a) Gordan, network derived from motif instances from position weight matrices from protein binding arrays followed by manual curation [31], (b) Harbison et al.'s ChIP-chip data considering the exponential (Harbison exp) and other conditions (Harbison other) separately [6], (c) A recent ChIP-chip data under normal (Venter 25C) and heat shock (Venter 37C) conditions from Venter et al. [9], (d) Yeastract, a public database comprising regulatory edges based on ChIP-chip, and factor knockout [32], (e) Rhee, high-resolution ChIP-exo neworks from Rhee et al for four transcription factors [29], (f) MacIsaac, a network which combined ChIP-chip data [6], and evolutionary conserved transcription factor motif instances to derive a regulatory edge between a transcription factor and a target gene [30]. While these networks are not perfect in reflecting the ground truth of the yeast regulatory network because ChIP-chip or -seq networks are condition-specific and the conditions do not overlap completely with the conditions from which we have mRNA data, an enrichment in these edges provides support of our inferred networks. Furthermore, the MacIsaac et al. network was used as the gold standard yeast regulatory network by the DREAM consortium [20]. A comparison based simply on the number of edges in the networks showed that MODNET inferred the most sparse network (**Figure 3A**), including 3,185 connecting 1,821 genes and 60 regulators, whereas the GENIE3 networks had the most edges (20,000 at top 20%). The size of the networks inferred by MODNET (3,185), MERLIN (6,319) and LINEARREGR (6,860) most closely matched the size of the MacIsaac network (4,153 edges).

Using edge-based measures we found that the network inferred by MERLIN had the highest fold enrichment with the different networks compared to the other methods (**Figure 3B**) (t-test 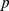-value <0.003). Interestingly, none of the inferred networks were enriched in Venter et al's network measured in exponentially growing cells, but exhibited enrichment in the network measured in heat shock stress, suggesting all methods can capture condition-specific edges to some extent, and are also internally consistent with each other. Comparisons using regulator-based measures showed that MERLIN's inferred network was as good or better than other inferred networks exhibiting overlap of ChIP-chip targets of as many regulators as any other method (**Figure 3C**). For module-based comparisons (**Figure 3D**), since true modules were not known, we used curated gene sets as modules. This included Gene Ontology Slim terms [33] and Yeastcyc bio-chemical pathways downloaded from the Saccharomyces Genome Database [34]. We treated the MacIsaac network as the true network and compared the number of regulator-module relationships from the MacIsaac network with those from the inferred regulatory networks. We found that in all measures MERLIN was better or as good as other methods with only a slight decrease in enrichment for the Ecocyc pathways. In all these measures the relative performance of MODNET was closer to the other networks compared to simulated networks.

As a final comparison, we asked whether the modules inferred by MODNET and MERLIN represented targets of specific TFs by examining each module for enrichment of a TFs' ChIP-based targets in the module. This analysis was possible only for MODNET and MERLIN which infer modules but not for any of the per-gene methods. We found both MODNET and MERLIN modules were enriched for the ChIP targets of a large number of transcriptions (**Figure 3A**, column 5). This suggests that the module information captured in both MERLIN and MODNET represent co-regulated sets of genes, and allows us to gain new insight into the module-level properties of networks that are not evident in the per-gene methods.

Overall, we find that MERLIN performs as well or better than other methods on different types of metrics. Per-gene methods did not reveal any module structure and thus assessing whether TF's targets were associated in modules was not possible. The per-module method, MODNET, performed poorly on edge-based metrics, but had better performance on the yeast regulatory network which had more genes (although we cannot rule out that simulated networks are not perfect). Thus MERLIN combines the strengths of both the per-gene and per-module network inference methods, inferring high quality reconstructions of individual regulatory edges, as well as high confidence target sets localized to specific modules.

### Dissecting yeast stress responses using MERLIN

**Figure 4:**
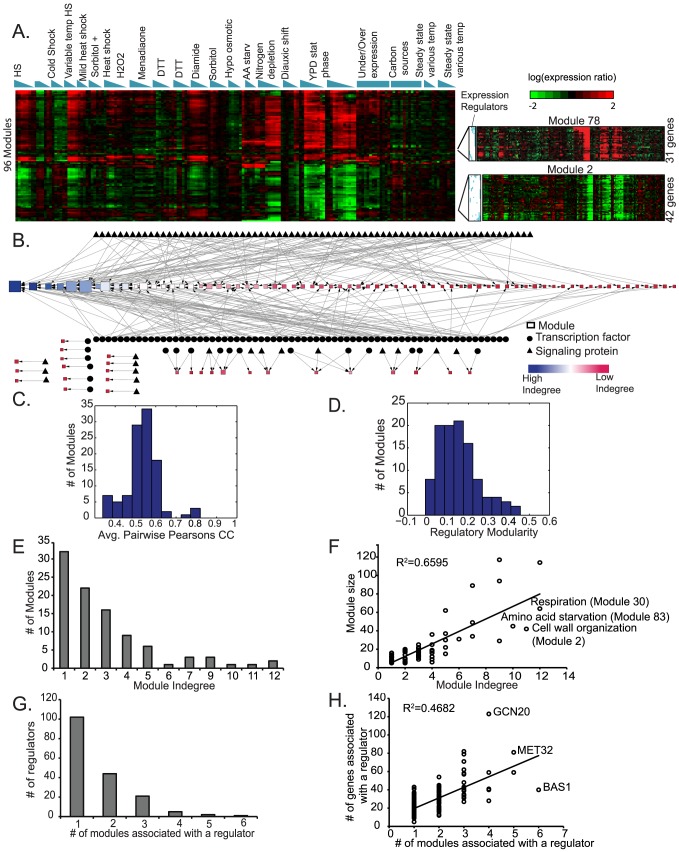
Global organization of yeast stress response network revealed by MERLIN. **A.** Major patterns of expression in each module inferred by MERLIN. Each row represents the mean of the expression profile of a module. The rows are ordered based on hierarchical clustering of the means of the modules followed by an optimal leaf ordering of the rows so that rows that are the most similar to each other are closest. **B.** Shown are the regulators and modules with edges from regulators to target modules (squares). The size of the module is indicated by the number of genes in each module. The color and the ordering of the module nodes is according to the number of regulators associated with each module. **C.** Histogram of average Pearson's correlation between each pair of genes assigned to a module. Majority of the modules have greater than 0.5 correlation suggesting genes in a module are co-expressed. **D.** Histogram of regulatory modularity of a module measuring the extent to which genes from the same module share predicted regulators versus between genes from different modules. High regulatory modularity suggests genes in the same module share more regulators than genes that are not in the same module. **E.** The distribution of the number of regulators per module **F.** Scatter plot of module size (number of genes assigned to a module) versus the number of regulators associated with a module based on enrichment of its predicted targets in the module. Module indegree and module size are linearly related (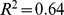). Outlier modules with more regulators than expected by a linear fit to the module size are indicated on the plot. **G.** Distribution of the number of modules associated with a regulator. **H.** Scatter plot of the number of modules associated with a regulator based on its predicted target set enrichment versus the number of target genes predicted to be regulated by the regulator.

We next used MERLIN results from the Gasch et al data [24] to study the regulatory network from a module point of view and to gain additional insight into the regulation of yeast stress responses. We focused our attention on 106 modules with five or more genes, which together encapsulated 80% of the genes in the original dataset (**Figure 4A,B**).

#### MERLIN identified tightly co-expressed modules with distinct regulatory programs exhibiting coherent biological function

First we asked whether MERLIN modules are indeed coherent groups of co-expressed and co-regulated genes by measuring: (a) the average Pearson's correlation for pairs of genes within a module (**Figure 4C**), (b) regulatory modularity (**Figure 4D**), which quantifies how different the regulatory programs are of one module compared to other modules (**Materials and Methods**). Correlation and regulatory modularity measures are always between −1 to 1 for each module, with a positive number indicating that genes in a module are tightly co-expressed and have distinct regulatory programs, respectively. We found that genes within modules indeed are tightly co-expressed (average Pearson's correlation >0.5 for 82% of modules) and have high regulatory modularity (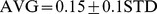), confirming that genes in a module share many more regulators with genes within the module than with genes not in the module.

Next, we evaluated whether modules are biologically meaningful by testing whether the modules were enriched for genes annotated with specific Gene Ontology (GO) processes [33] (FDR corrected hypergeometric test, FDR<0.05, **Table S1**), targets of transcription factors based on ChIP-chip assays or genes with upstream sequence-specific binding motifs. The majority of the modules (63 of 96) were enriched either for genes of the same GO process, ChIP-chip targets of TF, or genes with sequence-specific motif instances of a TF. This enrichment together with the high Pearson's correlations between gene expression profiles assigned to each module suggests that modules inferred by MERLIN are co-expressed gene regulons that capture biological meaningful relationships among genes in a module.

Using the inferred regulatory network and module memberships, we derived a regulator-module relationship network if the regulator's predicted “targets” (defined here merely as the genes whose expression was predicted by the regulators expression pattern) were enriched in a given module (FDR<0.05). This network connected 175 different regulators to one or more of 96 modules (**Figure 4A,B**). We found the modules varied greatly in size, ranging from 5 to 117 genes with a median size of 11 genes. For each module we computed a “module in-degree” defined as the number of regulators whose targets were significantly enriched in the module. The module in-degree grew with the module size for most modules (**Figure 4E,F**, Pearson's correlation of 0.81), which is significantly higher than what is observed in random clustering (average Pearson's correlation = 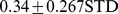, 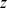-test 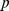-value <1E-33). This suggests that the high in-degree is perhaps because these modules have more regulators associated with them (but perhaps also influenced by ascertainment bias). However, there were exceptions to this trend. For example Module 2 (enriched for genes involved in cell-wall organization), Module 83 (amino acid biosynthesis genes) and Module 30 (respiration genes) had the largest number of associated regulators but only 42, 45 and 64 genes, respectively. The high number of regulators associated with these modules might represent complex combinatorial regulation or distinct condition-specific regulatory programs that operate under distinct situations, discussed more below. Other modules with the enrichments are available at our web-supplement http://pages.discovery.wisc.edu/~sroy/merlin.

#### Combinatorial regulation specifies distinct module expression patterns despite shared regulators

Several regulators were associated with a large number of genes in the MERLIN-inferred network, that is they had large out-degrees (e.g. Gcn20: 123 genes, Ime4: 82 genes, Met32: 81 genes) compared to the average (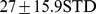), (**Figure 4H**). Because a regulator can influence gene expression differently depending upon the other regulators associated with these genes, we asked whether the targets of a given regulator were localized to a single module or whether they were distributed across different modules. First we identified the number of modules to which the regulator was associated (based on statistical enrichment of the regulator's predicted targets in the module, FDR<0.05). A large fraction of regulators (41.7%, 73 of 175) were associated with at least two modules, with a maximum of six modules linked to a given regulator (**Figure 4G**). The number of associated modules was only moderately correlated with the number of gene targets of a regulator (**Figure 4G**), e.g. the 40 Bas1 targets were distributed into six modules, while the 123 Gcn20 targets were distributed in four modules (**Figure 4H**).

There are two possibilities for why a regulator's predicted targets belong to different modules: either those modules are distinguished by dramatically different expression patterns across the diverse stress experiments, or they are distinguished by distinct set of predictive regulator sets even if their expression patterns are similar (but not identical). To systematically test these possibilities we computed the Silhouette index for each module, which measures how different a module's expression is compared to other modules. A positive Silhouette index for a module suggests that the expression of the module is distinct in expression from other modules. The Silhouette index was significantly higher than random clusters (KS test 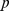-value <1E-38), however, the Silhouette indices for individual modules was low. In particular, while a third of modules had positive Silhouette indexes, the remaining two thirds of the modules had negligible or negative Silhouette scores indicating that the modules share some similarity in expression patterns. This observation in concert with the high regulatory modularity measure and high co-expression within each module suggests that distinct regulatory programs are associated with modules that are similar, but not identical, in expression pattern.

**Figure 5:**
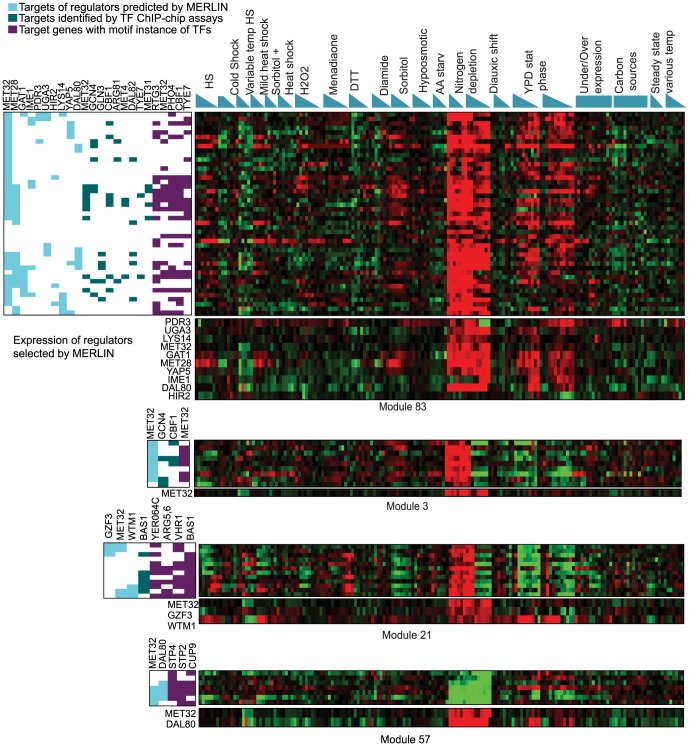
Amino acid starvation modules associated with Met32 and other amino acid bio-synthesis regulators. Modules predicted by MERLIN to be associated with Met32 exhibit distinct patterns of expression. Shown are four modules that each have Met32 as an inferred regulator based on gene expression. Cyan represents expression-regulators, teal represents ChIP-chip targets of regulators whose ChIP-chip targets are enriched in the module, purple represents targets that have a motif sequence of a regulator. Only regulators that are enriched in this module are shown. For each module, the heatmap is separated into the expression of the genes in the module and expression of the regulators selected by MERLIN.

An example of this situation is seen for the Met32 transcription factor, a key regulator of sulfur metabolic genes that was associated with several different modules. All of these modules were enriched for amino acid pathway genes, but the modules exhibited different expression patterns in subsets of the diverse stress experiments (**Figure 5**). Genes in Modules 83, 21, and 3 were characterized by strong induction during amino acid starvation, conditions under which cells attempt to generate their own amino acids [24]. However, the modules were distinguished under other conditions: whereas genes in Module 83 were also induced under long-term nitrogen and carbon starvation, genes in Module 3 were additionally induced in response to the oxidizing drug menadione. In contrast, Module 57 genes were strongly repressed under amino acid and nitrogen starvation - this module included specialized amino acid transporters that are known to be repressed upon general amino acid starvation [8].

Beyond expression, the modules were particularly differentiated in terms of the inferred regulatory networks. In addition to Met32, genes in Module 83 and Module 3 were associated with other regulators that are known to combinatorially interact in the sulfur regulatory network, including Met4, Met28, Met31 and Cbf1 [7]. In addition, many genes were associated with Gcn4, which induces these genes in response to general amino acid starvation [35]. In contrast, Module 21 was associated with Met32 and the Bas1 transcription factor, which regulates purine biosynthesis genes – indeed, this module was enriched for purine genes that are induced during amino-acid starvation along with amino acid genes. Finally, genes in Module 57 were associated with Met32 and the nitrogen-responsive regulator Dal80, and enriched for the known targets of the Stp2 and Stp4, which regulate expression of amino acid transporters. In this case, the expression of Met32 is predictive of the repression of the specialized amino-acid transporters in the module, which are functionally related to but anti-correlated with Met32 targets.

#### MERLIN identifies upstream signaling networks linked to stress-activated expression programs

**Figure 6:**
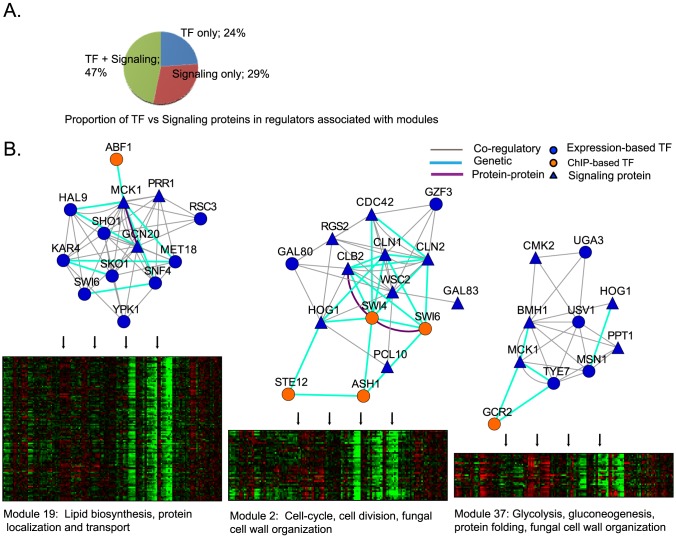
Interplay of transcription factors and signaling proteins in specifying the regulatory programs of modules. **A.** Shown are the fraction of modules that are regulated by TFs alone, signaling proteins alone or both **B.** Shown are the co-regulatory, genetic and protein-protein interactions between regulators associated with HOG1 associated modules. HOG1 is a protein kinase involved in osmotic stress and cell wall organization. HOG1 is predicted to be a regulator for Modules 2 and 37, and is known to be directly upstream of SKO1 which is predicted to regulate genes in Module 19. Co-regulatory relations are inferred between two regulators if they share common targets. Genetic and protein-protein interactions are obtained from BioGRID [66].

Many of the modules identified by MERLIN were associated with upstream signaling proteins as well as transcription factors. Of the 96 modules, 45 were associated with both transcription factors and signaling proteins while 28 were associated only with signaling proteins (**Figure 6**). In several cases, MERLIN identified subunits of signaling complexes (for example Cka1 and Ckb2 subunits of the CK2 kinase in Module 36, and Reg2 and Gac1 subunits of the Glc7 phosphatase in Module 39) or regulators responding to similar conditions (such as osmotic stress regulators Sho1, Mck1, Hog1, Ypk1, Msn1, and Sko1 in Modules 2, 19 and 37).

To more broadly study these relationships, we asked if the set of regulators selected for each module exhibited functional relationships. Indeed, we found several modules whose implicated regulators (either via MERLIN or ChIP-target enrichment) were enriched for genes whose proteins display more genetic or physical interactions among each other than expected by random (**Table 1**, **Materials and Methods**). This result strongly suggests the presence of functional relationships between implicated regulators.

**Table 1:** Yeast regulatory modules with regulators enriched with physical or genetic interactions.

Module ID	#genes	TF	Signaling	ChIP	Annotation
19	114	KAR4, RSC3, SNF4, MET18, SWI6, SKO1, HAL9	SHO1, GCN20, PRR1, YPK1, MCK1	ABF1	glycerolipid biosynthesis, protein transport and localization
2	42	SWI4, STE12, ASH1, SWI6, DIG1	GAL83, PCL10, CDC42, CLN1, WSC2, RGS2, CLN2, CLB2, HOG1	GZF3, GAL80	protein glycolsylation, fungal cell wall organization
83	45	PDR3, UGA3, LYS14, MET32, GAT1, MET28, YAP5, IME1, DAL80, HIR2		MET32, GCN4, GLN3, CBF1, ARG81, MET4, DAL82, TYE7, MET31	sulfur and methionine metabolism, serine and aspartate amino acid metabolism
68	13	ASH1	STE2, SIC1	SWI5, STE12, ACE2, MCM1, FKH2	cell wall organization and bioenesis, cytokinesis
100	7	KAR4	STE2	STE12, DIG1	response to pheromone, sexual reproduction
30	64	YHP1, GCN4, YFL052W, GSM1, HAP4, GAL80, WTM1, AFT2, ARR1	REG2, PCL7, FAR1, GAC1	HAP3, HAP2, SKN7, SIP4, HAP4, HAP1, MOT3, HAP5, AFT2, ROX1	purine metabolism and mitochondrial ATP synthesis, respiration, purine nucleotide metabolic process
31	22	GIS1, GCN4, BAS1	PPH3, YVH1		ribosome biogenesis, RNA metabolism
34	14	SWI5	CDC20, CDC5	SWI4, STE12, FKH1, SWI6, MCM1, FKH2, NDD1	cell division, cell cycle, M phase
36	96	SNF4, IME4, SKO1	RDI1, CKB1, CKA1, CNB1, ARP9, PLP25		protein transport and localization
37	29	UGA3, MSN1, TYE7, USV1	HOG1, BMH1, PPT1, MCK1, CMK2	GCR2, TYE7	glycolysis, gluconeogenesis
44	62	OPI1, MET18	GCN1,PPT1		protein localization and transport, RNA transport
53	15	RDS1, STB5, YAP1, YJL206C	SGD1	YAP6, MSN4, YAP1, MSN2, YAP7	alcohol dehydrogenase genes
72	13		SHP1, SLT2, CNB1	REB1, RPN4	proteolysis, ubiquitin dependent protein catabolism

Each row corresponds to a Module. The first row specifies the Module ID, the second column has the module genes, the third column has the TF regulators predicted by MERLIN, the fourth column has the signaling proteins predicted by MERLIN, the fifth column has regulators predicted based on ChIP-chip, and the last column has a summary of the Gene Ontology terms associated with each module.

One such example included Modules 2, 19 and 37 that exhibited somewhat distinct expression patterns but were associated with regulators from the Hog1 signaling pathway, which regulates cell cycle progression and the response to high osmolarity [36] (**Figure 6**). The regulators predicted for Module 2 also included several cell-cycle regulators, including cyclins Pcl10, Cln1, Cln2, and Clb2, G1-specific transcription factors Swi4 and Swi6, and Wsc2 and Cdc42, which respond to cell wall or cytoskeletal defects and were enriched for Hog1 signaling pathway genes (See **Materials and Methods**, hypergeomteric 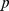-value <0.02). Indeed, Module 2 was enriched for cell wall genes including those regulated by the cell cycle. Module 37 was also associated with Hog1 as well as stress-activated transcription factors Msn1, Usv1, Tye7 and stress-activated kinases Cmk2 and Mck1 (the latter of which we found regulates many Hog1 targets upon osmotic stress (Gasch lab, unpublished). This network also included phosphatase Ppt1, recently shown to dephosphorylate Cmk2 [37], and the 14-3-3 protein Bmh1 - both regulate stress-activated transcription factors [38]–[40]. Module 19's members were moderately enriched for Hog1 signaling pathway genes, and was predicted to be regulated by Sho1 and Sko1, both acting downstream of Hog1 [36]. Other modules whose upstream regulators showed abundant interactions included genes involved in a wide variety biological processes including amino acid metabolism (Module 83), respiration (Module 30), and, protein modification, transport and localization (Modules 36 and 72). The enrichment of genetic interactions suggests that such signaling proteins are likely upstream regulators of transcriptional regulators associated with these modules.

### MERLIN identifies regulatory modules exhibiting waves of transcriptional changes during differentiation neural progenitor cells

To test our approach on another dataset, we applied MERLIN to infer transcriptional regulatory networks in a very different biological context: during differentiation of human embryonic stem cells to neural progenitor cells. Four time courses were available that represent the first seven or eleven days of differentiation from the pluripotent state (either ES or iPS cells) to states that represent early neural precursor cell types. Each cell line was treated by two different conditions to induce neural differentiation (**Materials and Methods**). The final states are not likely identical in all four time courses, but they share many characteristics and all are representative of early neural differentiation, so we concatenated them into a single dataset. To study the interplay between transcription factors and signaling proteins during differentiation, we included as regulators, transcription factors from a recent comparative study of human and mouse from [41] and proteins annotated as phosphatases and kinases from Uniprot [42]. After initial data pre-processing to remove unchanging genes (**Materials and Methods**), we gave as input to MERLIN 5670 genes and 823 regulators (535 transcription factors and 288 phosphatases and kinases). We focused on the high confidence MERLIN-inferred network of 4647 genes, 90% of which were organized into 94 modules, with at least 5 member genes, associated with 326 regulators. We examined these modules for biological function based on enrichment of genes annotated with Gene Ontology processes [33], genes annotated in pathways in the Molecular Signature Database (MSigDB, [43]), ChIP-seq targets of transcription factors from ENCODE [44], and motif instances of transcription factors in DNAse1 hypersensitive sites [45]. We discuss these results below (detailed module profiles and enrichment analysis are available from the web-supplement http://pages.discovery.wisc.edu/~sroy/merlin).

#### Transcriptional behavior during differentiation is captured by two large modules associated with ES state maintenance and neural differentiation

**Figure 7:**
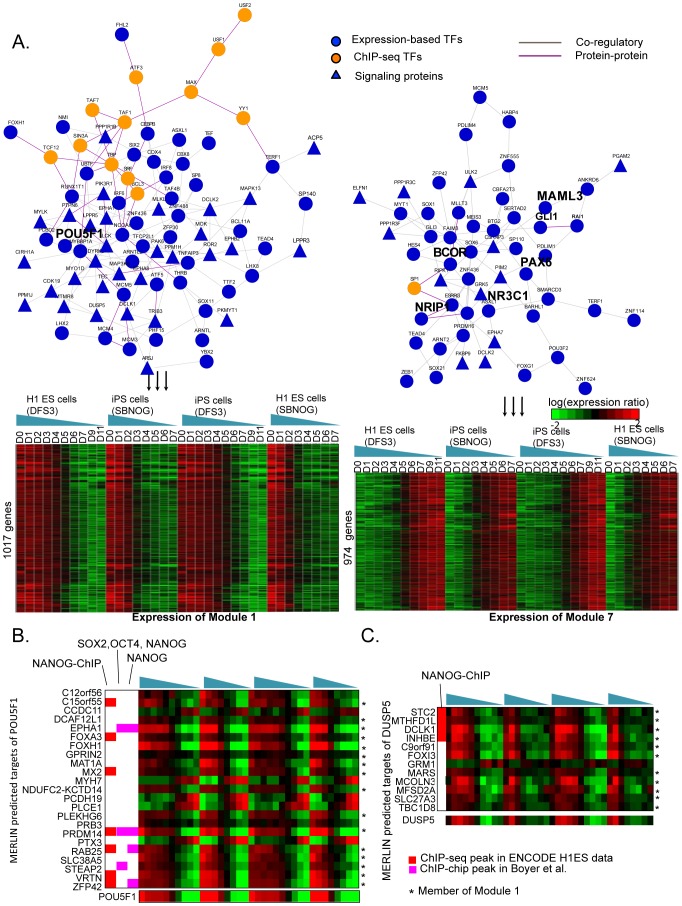
Application of MERLIN to differentiation time course of human ES to neural precursor cells identifies two large modules with opposite patterns of expression. **A.** Shown are the two modules, Modules 1, and 7, that exhibit characteristic temporal patterns of expression together with their predicted regulators from MERLIN and regulators whose ChIP-seq targets are enriched in the module. Known pluripotency maintenance regulators (POU5F1), and predicted neural fate driver genes are shown in larger fonts. **B.** Predicted targets of POU5F1 using MERLIN. * denotes membership in Module 1, which we associate with maintenance of ES state. MERLIN can infer both repressive and activating relationships between TF and target genes, e.g. CCDC11 and POU5F1. We also show ChIP-seq (Red column, NANOG-ChIP, [44]) and ChIP-chip datasets (Magenta columns, SOX2-OCT4-NANOG targets from Boyer et al., [71]) **C.** Predicted targets of DUSP5 using MERLIN. Some DUSP5 targets are also occupied by NANOG transcription factor.

The majority of the transcriptional changes are captured in two large modules, Module 1 with 1177 genes, and Module 7 with 974 genes (**Figure 7A**). Genes in Module 1 were highly expressed early in the response (up to day 4) and repressed after that. Genes in this module were enriched for general metabolic processes (carboxylic metabolic and oxoacid metabolic process), growth, and cell cycle (G1/S transition) (**Table S2**). In contrast, genes in Module 7 were associated with an opposite pattern of initial repression in the earlier time points (up to day 4) and upregulation at later time points. Several lines of evidence, in addition to the opposite temporal dynamics, suggested that Module 1 is associated with the maintenance of the ES pluripotent state, whereas Module 7 is associated with neural fate specification. **First**, genes associated with maintenance of the ES state such as POU5F1 are represented in Module 1. **Second**, Module 1 is enriched for ChIP seq-based targets of several factors, obtained from the ENCODE project [44], associated with the maintainance of pluripotency (NANOG, YY1 [46], [47], GABPA, ATF3, FDR<4.91E-4) and components of general transcription machinery (TAF1, TAF7, TBP, FDR<1E-12). Knockdown of several TFIID components was recently shown to interfere with pluripotency maintenance and obstruct reprogramming of differentiated cells to induced pluripotent state [48]. **Third**, 16 of POU5F1's 23 MERLIN predicted targets (**Figure 7B**), and 11 of DUSP5's 12 targets (**7C**), two factors critical for pluripotency [49], were present in this module, further asserting that this module represents the ES-state maintenance genes. **Fourth**, Module 7's genes are associated with neural-specific functions such as nervous system development, neurogenesis (FDR<0.05) suggesting these genes are neural lineage specific genes. **Finally**, using a recent set of lineage specific genes, we found Module 1 to be enriched for the ES-specific and Mesendoderm, an early differentiation time point, genes (FDR<1E-51), and Module 7 to be enriched for the neural lineage-specific genes [50] (FDR<1E-75), further supporting the different embryonic stages associated with these modules.

#### Linking signaling proteins with transcription factors in ES cell differentiation

MERLIN predicted numerous regulators associated with these modules including both transcription factors and signaling proteins. As in the yeast stress response dataset we asked if these regulator sets were enriched for protein-protein or genetic interactions. Regulators associated with Modules 1, 7 and 70 are indeed enriched for protein-protein but not genetic interactions, suggesting more direct interactions among these regulators. In particular, regulators predicted by MERLIN for Module 7 identified several striking connections between major signaling pathways to transcription factors associated with cellular differentiation in different lineages. For example, MERLIN predicted GLI1, GLI3, MAML3 and RAI1 as regulators associated with Module 7, which interact physically [41] and are associated with the Hedgehog signaling pathway (GLI1, [51]), Notch signaling pathway (MAML3, [52]) and retinoid acid signaling pathway (RAI1, [53]). Hedgehog signaling in the floor plate during neural development is mediated by GLI1 [51]. Notch and retinoid acid signaling play important roles in neural development [54], [55]. The MERLIN identified regulators also include the transcription factors, MLLT3 myeloid differentiation [56] and BCOR associated with tissue homeostasis, [57] that exhibit protein interactions with nuclear receptors (NR3C1 [58], and NRIP1) which interact with retinoids to regulate cell differentiation [59].

#### Fine-grained modules associate oncogenes as potential drivers of neural differentiation

**Figure 8:**
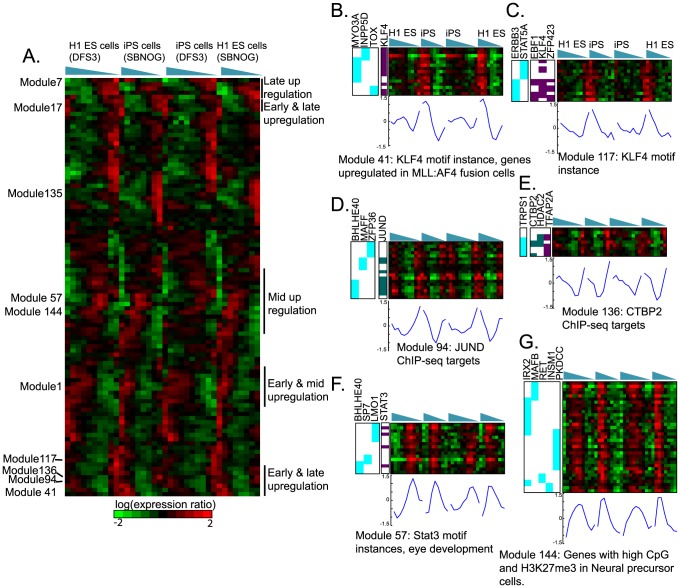
Modules expression patterns inferred by MERLIN on the human ES cell differentiation time course into neural progenitor cells. **A.** Each row corresponds to the mean expression profile of one module. The rows are ordered based on hierarchical clustering of the means of the modules followed by an optimal leaf ordering of the rows so that rows that are the most similar to each other are closest. This ordering enables us to see a gradual change in the different temporal dynamics captured in the MERLIN modules. **B–G.** Selected modules associated with complex temporal patterns. Cyan represents targets (rows) of regulators (columns) predicted by MERLIN, Teal represents ChIP-seq targets, and Purple represents presence of motif instance of a transcription factor ±2 kb around the Transcription Start Site (TSS) of a gene. **B–E** Modules associated with upregulation at the beginning and end of the time course whose members are also associated with oncogenes such as KLF4, MYC, JUND, and CTB2. **F–G** Modules associated with initial and late downregulation. **G.** Shown are the genes of module 144 which is enriched for genes exhibiting high CpG density in promoters in Neural Progenitor cells as annotated in MSigDB [43].

While the majority of the transcriptional response was captured by modules 1 and 7, we found several smaller modules that exhibited patterns of more complex temporal dynamics (**Figure 8B–E**). This included modules that exhibited up regulation at both early and late time points (Modules 117, 118, 41, 136, 94, **Figure 8B–E**). Several of these modules (Modules 93, 131) were also enriched for gene sets associated with skin tumour (Module 131, FDR<0.002) and prostate cancer (Module 93, FDR<0.03) obtained from Molecular Signature Database (MSigDB [43], **Table S3**). Interestingly some of the regulators associated with these modules based on ChIP-seq, DNAse1 filtered motif instances [45], or MERLIN are oncogenes (JUND, KLF4, MYC CTBP2, FDR<0.05). KLF4 and MYC are often used to reprogram cells back to the ES state [60]. It is conceivable that oncogenes are important both for early and later differentiation events. Oncogenes are highly upregulated during the early stages of axolotl limb regeneration presumably to allow reorganization of transcriptional programs [61].

Another set of modules exhibited an initial and late downregulation and an upregulation at intermediate states, opposite of the above modules (**Figure 8F,G**). One such module, Module 144, was enriched for an MSigDB gene set associated with high CpG density and the H3K27me3 mark in promoters (FDR<0.02). It has been shown that high CG and histone marks are associated with genes that are active in earlier developmental stages [**50**]. Finally, there were some modules that exhibited very delayed upregulation (Module 135), and were predicted to be under retinoic acid control. Retinoic acid signaling is thought to be involved in many aspect of development including neural differentiation [55].

In summary, using MERLIN we identified both regulators and modules describing different temporal patterns of transcriptional behavior. Several of the modules were associated with signature patterns observed in cancer cells or were enriched for motif targets of oncogenes, which can be tested as drivers of neural differentiation.

## Discussion

Per-gene methods for reconstructing gene regulatory networks do not exploit modular organization of regulatory networks, whereas per-module methods do not have the resolution for capturing gene-specific regulatory information. Our novel approach, MERLIN, combines the strengths of both per-gene and per-module network inference methods by inferring the regulatory programs for each gene and also capturing the modular structure of the regulatory network. On both simulated and real expression data MERLIN correctly inferred precise regulatory programs associated with individual genes and also captured modular organization of regulatory networks that provide new insight into the dynamics and regulation of transcriptional responses in our studied biological contexts.

### MERLIN strikes a balance between gene-specific and module-level regulatory information in the same algorithm

We found that while GENIE3, a state-of-the-art per-gene method performed well using edge-based metrics, when applied to yeast, it did not perform as well on module-based measures. In contrast, MODNET, a state-of-the-art module-based method, performed poorly using edge-based measures, but performed better on module-based measures. This improvement in performance was due to the module information allowing us to restrict ourselves to targets that are co-expressed in a module and thereby exhibit coherent function. Indeed identifying co-expressed sets of genes is a pre-requisite to identifying meaningful *cis*-regulatory elements enriched with a set of genes. A per-gene method does not provide such information making it difficult to identify the regulatory modules comprising genes sets that are co-regulated by multiple regulators. MERLIN's strengths are in its ability to combine the complementary advantages of both classes of methods. An additional advantage of MERLIN is that it is based on a probabilistic graphical model, which infers network parameters in addition to structure. Our preliminary work on assessing expression prediction on a holdout set shows that MERLIN outperforms LINEARREGR on more genes than it is outperformed, suggesting that incorporating the module constraints can also benefit the predictive power of the model (**Figure S2**).

### MERLIN is widely applicable to biological processes with different dynamics

Our application of MERLIN to the yeast stress response data and the human embryonic stem cell differentiation data reveals its ability to dissect the transcriptional regulatory programs especially in large datasets that measure diverse conditional responses. In particular, in the human embryonic stem cell differentiation data, the module with genes that are up-regulated in the later time points but not in the earlier (ES) time points had little or no enrichment in the ChIP-seq targets. Incidentally, ChIP-seq datasets were generated in the H1-ES (human embryonic stem cells) cell line, and the modules in which there was enrichment comprised genes that were most expressed in the earlier time points that reflect a more ES-like state. MERLIN therefore captures context-specific regulatory interactions. Such interactions are most enlightening for modules exhibiting induced expression in conditions or tissues that have not been studied in great detail, perhaps due to under-sampling of these biological responses. Even in the yeast stress response data, which is a very well studied dataset, we derived new insight into the role of HOG1 and the downstream modules that might be regulated through intermediate transcription factors as we discuss below.

Our ability to capture these regulatory networks likely centers on the inherent feedback and feed-forward loops in eukaryotic transcriptional responses: genes encoding signaling proteins are often themselves targets of the pathways they encode. In other cases, genes encoding regulatory proteins (especially negative regulators) are augmented in anticipation of their future need; nonetheless, their expression patterns remain predictive of physiologically related genes. These features are likely to be common to many different responses across diverse organisms.

An important difference between the yeast and the human dataset was the number of biological conditions the genes were measured in. In particular, in yeast we had more than a dozen environmental perturbations whereas in human we had four relatively similar kinds of perturbations. While the relatively uniform nature of expression dynamics in this dataset enables us to identify the major patterns of expression as two large modules, adding more diverse perturbations can help us identify smaller fine-grained modules as in the yeast dataset that are easier to interpret biologically and for follow-up studies with smaller functional assays.

### Extensions to MERLIN

There are several directions of future work associated with MERLIN. An immediate step is to more accurately model expression levels from next-generation sequencing data by considering conditional negative binomial or Poisson distributions [62]. MERLIN can be easily applied to other regulatory genomics datasets including global chromatin states that are becoming increasingly available using approaches similar to Marbach *et al* [63], taking a weighted union of different MERLIN inferred networks. Another, perhaps more principled, way of integrating such datasets would be through extending MERLIN's prior to incorporate more detailed features of the promoter architecture of a gene such as sequence-specific motifs and nucleosome occupancies. On a related note, it is possible to combine different types of proteomic datasets within the MERLIN framework, e.g. using measured protein levels of transcription factors and signaling proteins and existing physical interactions to predict the mRNA levels of genes. Such extensions can likely better capture the transcription factors based on ChIP-chip than what we are able to do based on expression alone that might miss changes such as post-translational modifications on the regulators. Specifically focusing on temporal dynamics, one direction of research is to predict the expression state based on observations made at a previous state, which can model delays in transcriptional responses. MERLIN can also be extended to capture non-linear relationships between a target and a regulator expression profile. This can be done using a random forest regression approach which has the additional advantage that the trees can be gleaned to identify combinatorial rules of regulatory logic or through an S-system model that models both non-linear and temporal dynamics [16].

The increasing abundance of environment-specific, tissue-specific and disease-specific transcriptional profiles, especially for poorly characterized organisms, makes our ability to infer regulatory networks especially important. Approaches such as MERLIN that identify the gene-specific regulatory information for individual genes, while revealing the global modular organization of regulatory networks can significantly advance our understanding of wiring and combinatorial regulation of transcriptional responses governing cellular states.

## Materials and Methods

### Details of the MERLIN algorithm

The MERLIN approach is based on a probabilistic graphical model of network inference where the goal is to infer regulatory networks by maximizing the likelihood of observed expression data given a network structure [14], [25]. We use a similar notation as described by Segal et al [14]. Let 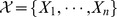 denote the set of random variables, each taking a value 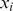 from the domain 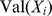. Each variable, 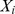 in turn represents the 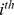 gene or a regulator and there are total 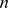 genes. Thus 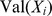 is a possible expression level of a gene measured in a microarray or from an RNA-seq experiment (See data pre-processing for more details of what we mean by level). A subset of variables 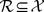 where 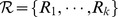 denote the candidate regulators. We assume that we have a set of gene expression measurements for the 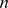 genes denoted by 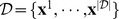, where 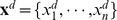 denotes the joint assignment of expression values for all 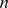 genes in the 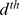 sample.

The model that MERLIN learns has three components: 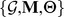. 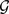 denotes the unknown regulatory network of interest describing the regulatory relationships between genes and regulators. Note that a regulator itself is also a gene and we can infer its regulators as well. 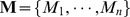 denotes the set of module memberships of each gene, where 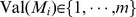 and 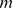 denotes the total number of modules. 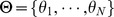 denotes the set of parameters, with each 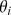 denoting the parameters of the conditional distribution, 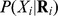, of a target gene 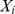 and its regulators, 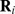. Several forms are possible for 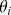. For example if we assume 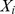 is a linear combination of the levels of the regulators, 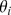 is the set of regression coefficients for each regulator selected for a gene. If we want to capture non-linear relationships, we can use a regression tree, where 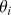 would represent a collection of means and variances of a target gene at each leaf node. As we discuss in the score below, we assume that 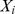 and its regulators are distributed according to a multivariate Gaussian.

#### Score in MERLIN

Given 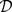, the set of candidate regulators 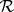, and an initial assignment of modules 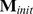 our goal is to infer the regulatory network 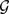, 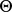 and new module assignments 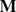. Note the number of modules specified in 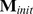 might not be the same as the number of modules specified in 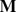. To infer the unknowns in our model we use a score based approach in a Bayesian framework. We treat 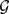, 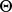 and 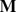 as random variables and we wish to find the posterior probability of these unknown variables. Our score 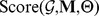 is proportional to the posterior probability of these unknowns 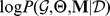. By Bayes rule 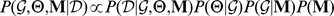. Thus our score is
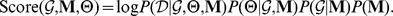
Here 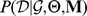 is the data likelihood, and 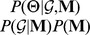 corresponds to the model prior. We assume that 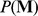 is a uniform prior and does not influence the score. Given a 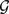 we set 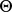 to its maximum likelihood settings. The quantity that we need to define is 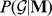, the graph prior. It is in this quantity that we incorporate our module constraint. We write 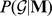 as a product over regulatory edges that are present (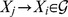) and edges that are absent (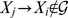) in the graph 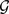, that is 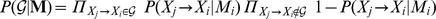, where 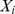 corresponds to a target gene and 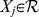 is a regulator. The probability of an edge 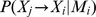 is written as a logistic prior 
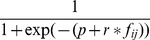
, where 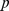 and 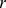 are hyper-parameters and 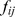 is an edge-specific feature measuring the extent to which regulator 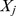 regulates other genes in 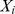
's module, 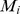. 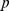 controls the sparsity of the graph and 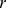 controls the affect of the module prior. For any edge 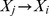 and fixed 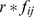, the more negative 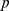, the smaller the value of 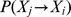, whereas the larger the value 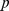 the higher the value of 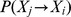. For a very negative value of 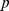, the data likelihood has to improve by a much greater margin for an edge to be added into the network. Thus, the more negative the 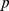 the more sparse is the network.

Given a module assignment and a graph at a particular learning iteration, 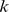, 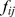 is the ratio, 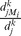, where 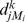 is the number of predicted targets of 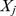 in 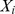
's module in iteration 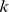, and 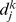 is the total number of predicted targets of 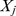. We rewrite the graph prior part of the score as a product over the regulatory edges associated with each variable, 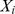:
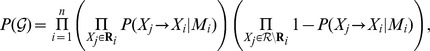
where 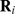 is the set of regulators associated with gene 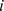 in 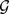. We denote the term inside the product as 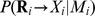 to denote the subgraph induced by 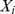 and it's regulators, 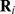.

The likelihood 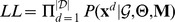 also can be written as a product over each variable, where each term in the product specifies the contribution of a variable to the overall likelihood part of score. The graph 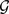 itself is a dependency network which allows us to capture cycles, thus 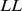 is the pseudo likelihood. 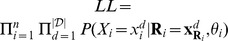, where 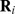 is the set of regulators associated with gene 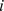 in 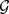, 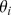 are the parameters associated with the conditional distributions 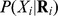, and 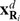 are values assigned to 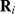 in the 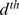 data sample. We denote the likelihood contribution of each variable as 
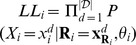
. We assume that 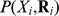 are distributed according to a 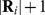-dimensional Gaussian. This Gaussian is estimated for each 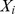 and candidate 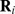 pair. We convert the joint Gaussian into a conditional Gaussian with parameters, 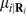 and 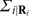 as described in Lauritzen [64]. Thus 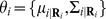. We have found that our approach of using the multivariate Gaussian works better than the standard linear regression approach because it takes into account the dependencies among the co-variates. Because both the likelihood and structure prior decompose over individual variables, we can write the score over the full graph as sum over variables: 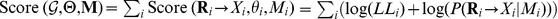.

#### MERLIN learning algorithm

The algorithm (**Algorithm 1** in **Text S1**) begins with a set of modules 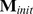, which are typically defined by an expression-based clustering step. It then iterates over two steps (**Figure 1**): (a) identifying the regulators for each gene given the current module assignments, and (b) re-inferring the modules using both co-expression and the inferred regulators for a pair of genes. It repeats these two phases of the algorithm until convergence. During the regulator identification step, the algorithm grows the regulator set of each gene based on the improvement in prediction error of expression of a gene, subject to a structure complexity prior that penalizes too many regulators.

To infer the 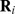 for each gene we directly estimate the Markov blanket of each gene, 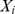, defined as the set of variables in 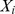
's immediate neighborhood and which render 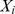 independent of all other variables. We constrain the members of the Markov blanket to the set of regulators 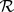. To identify 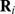 we use a greedy approach that grows the regulator set (initially empty) for each gene 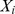 by adding the next regulator which when added gives the greatest score improvement. In particular, we optimize 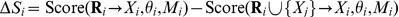, where 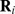 is the current regulator set or Markov blanket of 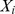. We add 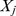 only if we observe an improvement in score. For each gene we begin with an empty set **R** *_i_* = Ø, and grow the set by considering candidate regulators 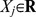, computing the score improvement on adding each 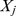 and picking the 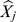 that has the maximal score improvement. To enable efficient computation of the graph prior, we pre-compute this quantity for an empty graph, and update it as we add edges during the learning process.

Once we finish the regulator selection phase for all genes, we have for each gene, 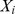, the weight 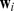 of regression coefficients for each 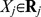. We revisit the module memberships of all genes, 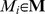, by computing a distance metric for all pairs of genes, 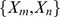, 
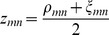
, where 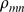 is 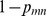 and 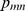 is the Pearson's correlation between 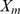
's expression 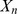
's expression, and 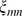 is the regulatory similarity of 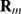 and 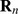 and is given by a modification of the Jaccard coefficient that takes into account the sign and magnitude of each element in 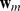 and 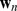. In particular, let 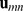 denote the indices of regulators associated with 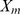 and 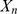, and that 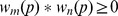, then 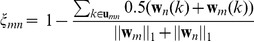. Here 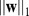 implies the L1 norm of vector 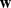.

Once we have defined the pairwise distance metric for each pair of genes we use a hierarchical clustering algorithm to define modules, stopping when the smallest distance between any two modules is greater than a given threshold, 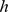. To efficiently do hierarchical clustering, we use the min-heap datastructure and merge nodes only if their distance is less than the threshold 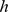. This approach of defining modules allows us to use the data to define the number of modules, and is not dependent excessively on the initial set of modules. When the algorithm converges, we have for each gene the module to which the gene belongs, as well as its regulator program (both the regulators as well as the parameters associated with each regulator). These steps are repeated until no more moves on the network structure changes gives a significant improvement in the likelihood score.

### Software availability

The MERLIN code and additional results on the Gasch stress data are available as a web-supplement at http://pages.discovery.wisc.edu/~sroy/merlin.

### Sensitivity of different parameters to performance of MERLIN

There are three parameters in MERLIN that need to be specified: (**1**) 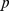 controls the number of edges in the network, that is the overall sparsity of the network, (**2**) 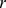, controls the extent of modularity in the network, (**3**) 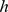 is the cutoff for deciding at what point we must stop the hierarchical clustering of the modules. To determine how the parameters influence the final structure, we carried out an extensive simulation experiment on networks of different sizes 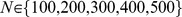 genes, and different modular structures. We used GeneNetWeaver (GNW) [27] to generate simulated expression data. We generated the structure of these networks outside of GNW in order to impose different extents of modularity by controlling a parameter 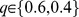. We partitioned the data into random non-overlapping modules, which included both a set of target genes and a set of regulator genes. For each edge we used 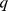 to probabilistically determine whether a regulatory edge would be added between a target and regulator gene with the same module membership, or different modules. Thus a higher value of 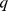 would favor greater regulatory modularity.

To assess the quality of the inferred network structure, we used F-score, which is defined as the harmonic mean of precision and recall. Precision in turn is the ratio of the true positives to the number of edges inferred, and recall is the ratio of the true positives to the total number of edges in the true network. The F-score gives us a single number that assesses the quality of the inferred network. The closer F-score is to 1, the better the performance. We also computed module statistics to assess the size and coverage of genes in each module: (a) number of good-sized modules, where a good-sized module must have at least 5 genes, (b) percentage of total genes that are in the good-sized modules. Ideally we would be able to include as many genes as possible in these good sized modules without losing biological coherence.

We found that the most important parameter that affected F-score was 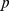, which controls the total number of edges in the network (**Figure S3, S4**). This was true for both the high modularity (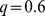) and low modularity (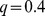) networks. For all networks of different sizes the optimal value as determined by the best F-score was 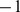. We also tried to pick 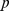 based on the cross-validation error, and picked 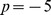 to have the lowest cross-validation error, but had considerably lower performance as measured by F-score. For a fixed 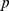, the other parameters had less affect on the F-score. The value of 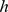 controlled the number of modules, with small values of 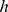 producing too many small clusters (<5 genes) and large values of 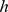 producing very large clusters (**Figure S5**). In general, the optimal value of 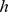 ranged from 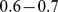, regardless of low or high modularity networks. Finally for a given 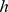, higher values of 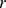 parameter tended to increase the number of good sized modules (at least five genes) and also the coverage of genes in these modules. The value of 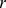 also affected the structure recovery. In particular for 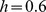 for the network with 500 nodes, a value of 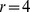 to 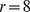 had a greater preference of low 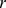 for the low modularity networks. This suggests that this parameter can effectively capture modular networks, however not very high values of 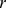 are required to do so.

Finally, we studied the effect of the 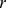 parameter on the modularity of the network (**Figure S6**). Because the networks we infer are directed regulatory networks, standard measures of modularity which are defined for undirected graphs were not sufficient. We defined a measure of regulatory modularity (defined below) that measures the extent of shared regulators of genes in a module compared to shared regulators with genes outside a module. We find that as we increase 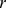, the estimated modularity of the network increases, but asymptotes after 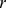 = 8.

### Data pre-processing

We applied MERLIN to two transcriptomic datasets, one in yeast [24], and one in human (Manuscript in preparation, some of the data was released in [50]). The yeast expression data was obtained from Gasch et al [24] and comprised 173 microarray measurements. The data was pre-processed by Segal et al, to remove genes that did not change significantly producing a total of 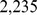 genes of which 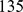 genes were signaling proteins and 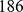 were transcription factors. We replaced missing values of a gene with its mean from other samples where its expression was available.

For the human data, we had RNA-seq read counts from four time courses, two each for two cell lines: human ES line H1 and human iPS cell line DF19.7. We assembled these reads into a per gene count using RSEM [65] and transformed the counts, 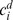 for each gene 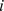 in the 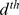 sample as 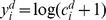. Next we computed the mean of each time course and subtracted the mean. Thus each expression level in the RNA-seq data, 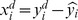, where 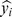 denotes the mean from a time course. The data were thus zeromean transformed for each time course separately. While this transformation does not account for the over-dispersed nature of RNA-seq data, we found that clustering the RNA-seq data using a Gaussian mixture gave us good performance as measured by Gene Ontology enrichment. Extending MERLIN to handle the “count” nature of RNA-seq data to reconstruct networks is an area of future work. After this we filtered out genes that changed less than ±1 in all time points. This produced a total of 5670 genes, of which 535 were transcription factors and 288 were either a kinase or phosphatase as annotated in Uniprot.

To apply MERLIN to each of these data sets we started with five random initializations. For the yeast data we split the data into five equal folds and learned models on four fifths of the data, and repeated this five times. A high confidence network was that which had an edge in three of the five random initializations. For the human data since we did not have as much data as in yeast, we used the entire data, but generated different MERLIN networks by starting with five different random initializations of clusterings 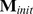. We created a high confidence network by considering edges that were present in all five random initializations. MERLIN was applied on the different datasets using 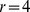, 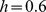 and 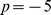. We selected these settings based on our experiments on simulated data where we found that the optimal values of modularity ranged between 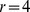 to 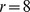.

### Regulatory modularity

We defined regulatory modularity for a set of modules, 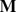 and a regulatory network 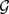 in the following manner. First, for any pair of genes, 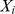, 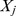 in the modules, we compute a regulatory similarity, 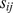 as 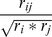, where 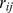 is the number of shared regulators between 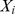 and 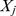, and 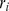 and 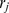 are the number of regulators for 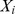 and 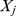 respectively. Regulatory modularity for a module 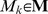 is defined as 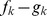, where 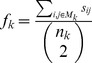 where 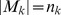, and 
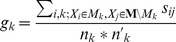
, where 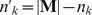. 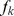 thus measures the within module regulatory similarity, and 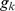 measures the regulatory similarity between genes in module 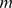 and all other genes. The denominators serve to normalize the 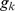 and 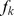 measures such that they are always between 0 and 1. The regulatory modularity for a module ranges between −1 and 1.

### Enrichment of genetic and protein-protein interactions

For each module with 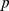 regulators (including ChIP-enriched regulators), we extracted the number of genetic interactions from the BioGRID database [66]. Next from our candidate set of regulators, we extracted a random set of regulators of size 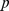 and also obtained the number of genetic interactions among proteins in the random set. We repeated the random set selection 100 times, and estimated a mean and standard deviation on the number of edges expected by chance. We next computed a 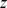-score using this background distribution. We considered a regulator set to have significant number of interactions if the 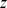-score was greater than 1. We repeated this process for protein protein interactions as well.

### Comparison of network inference methods based on edge and module-based metrics

We compared the performance of MERLIN on simulated networks to the performance of three other algorithms using edge-based, regulator-based and module-based metrics. These comparisons were done on simulated ground truth networks where the true networks were known. We used GeneNetWeaver (GNW) to generate simulated expression data [27] for networks of 100, 200, 300, 400, 500 and 1,000 nodes. GNW takes networks as inputs and uses a stochastic differential equation model to generate expression data. To generate data from GNW we used a similar strategy as was used in the DREAM project on network inference comparisons [20]. A set of measurements for all nodes was the steady-state values reached when the system was perturbed by simulating a gene knockout. All genes were knocked out, one at a time, producing datasets of size 100, 200, 300, 400, 500 or 1,000 measurements for the networks of different sizes.

#### Edge-based metrics for comparing the network inference algorithms

We first examined the agreement between the ground truth networks of different sizes to the inferred networks using standard precision-recall measures [28]. We computed the area under the precision-recall curve (AUPR) for each method, and used this as a metric to compare different methods on different networks. The AUPR ranges between 0 and 1, and the closer it is to 1, the better the inferred network in terms of the true edges recovered, and the false edges not inferred. As AUPR requires a continuously varying quantity we generated 100 random subsets of the data and computed an edge confidence using networks inferred on these 100 subsets.

In addition to AUPR we also compute the significance of overlap of edges between true and inferred edges using a hypergeometric 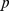-value and a fold enrichment. In fact, on the yeast network, AUPR was not informative due to the large number of “false” edges. The fold enrichment is defined as a ratio 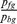 (also used in [63]), estimated from the same numbers used to estimate the hypergeometric 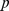-value. Namely, 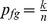 and 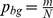, where 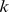 is the number true positive edges, 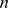 is the total number of edges inferred, 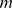 is the total number of edges in the true network, and 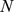 is the total number of edges possible, when restricted to common regulator and target nodes in the inferred and true networks. The fold enrichment gave us a single measure to compare both the simulated networks as well as the yeast network.

#### Regulator-based measures

In addition to edge-based comparisons, we developed measures that compare the inferred networks based on each regulator's target sets. This is done by considering one regulator at a time. For each regulator, 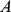, we ask whether its targets in the true network significantly overlap with its targets in the inferred network. Significance of overlap is assessed based on the hypergeometric distribution followed by a Benjamini-Hochberg correction for multiple hypothesis testing. We use FDR<0.05 to determine whether a regulator's predicted and true targets significantly overlap.

#### Module-based measures

To assess regulator-module relationships in the simulated datasets we used the modules that were part of the ground truth simulation. We generated the “true” regulator-module graph by asking what regulators' targets were significantly over-represented (hypergeometric 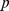-value with FDR correction, FDR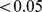), in each module using the “true” network. Such regulators were considered as the true regulators of the module. Next we asked, using the inferred networks, what regulators' targets are significantly overrepresented in the modules of the simulation. These gave us the predicted regulator-module relationships. We measured precision (defined as the fraction of predicted regulator-module relationships that were obtained from the true network), recall (defined as the fraction of true regulator-module relationships), and an F-score to summarize the precision and recall.

Our module-based measures on the yeast expression data used modules from GO slim annotations [33], or from the Yeastcyc pathways downloaded from the Saccharomyces genome database [34]. We next asked what transcription factors with ChIP-chip data identified in the MacIsaac et al network were enriched in each of these modules. A regulator whose ChIP-chip targets are enriched in a module is said to be associated with a module giving us “true” regulator-module relationships. Next for an inferred network we asked which regulators' predicted targets were enriched in each of these modules producing predicted regulator-module relationships. We computed both F-score and the 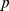-value from a hypergeometric test of overlap between the true and inferred regulator-module relationships. We report the hypergeometric test 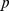-value as this was more sensitive to the differences between the algorithms on the real data. We generated our Regulator- and Module-based scores on the MacIsaac et al network only as this was the gold standard in the recent DREAM competitions of network inference methods [20].

### Enrichment analysis of modules using Gene Ontology and transcription factor binding sites

To assess the biological meaning of modules inferred by MERLIN we used gene set enrichment analysis of various modules using the hypergeometric test followed by an FDR correction method of Benjamini and Hotchberg. We considered only modules of size at least 5 genes. These gene sets included genes annotated with a Gene Ontology process [33] (yeast and human), ChIP-chip or ChIP-seq targets of transcription factors (yeast [30] and human [44]), gene sets from Molecular Signature Database (MSigDB, humans [43]), or genes with motif instances in DNase I hypersensitive sites (DHSs) [45]. To assess the association of Modules 2, 19 and 37 in the Gasch stress data we obtained a curated Hog1 signaling network from Tiger et al. [67] and combined it with a curated gene set from Gasch lab (unpublished), and asked whether any module members or the regulators of the module were enriched in this list. Based on a hypergeometric test of overlap we found regulators of Module 2 to be significantly enriched (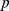-value <0.02), and a lower stringency of (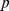-value <0.3) for the other modules. Module 19's regulators were also enriched for genes in this list (hypergeometric 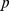-value <0.13).

To map ChIP-seq and DNAse1 sites in the human dataset we focussed on ±2000 bps of the transcription start site (TSS) of a gene, where the TSS coordinates were obtained from Gencode10 as used in the ENCODE project [68]. Motifs were obtained from Jaspar [69], and DHSs were obtained from Thurman et al [45], downloaded from http://ftp.ebi.ac.uk/pub/databases/ensembl/encode/integration_data_jan2011/byDataType/openchrom/jan2011/combined_peaks/. To find genes with motif instances of a transcription factor we used the Finding Individual Motif Occurrences (FIMO) from MEME suite [70] to scan the DHSs with a q-value <1E-5. To map a gene to a ChIP-seq peak of a transcription factor we obtained peak calls from the ENCODE project from http://ftp.ebi.ac.uk/pub/databases/ensembl/encode/integration_data_jan2011/byDataType/peaks/jan2011/spp/optimal/hub/, and associated a gene to the transcription factor if its peak was within ±2000 bps of the TSS of the gene.

## Supporting Information

Figure S1 **Edge-based comparisons on simulated networks of different sizes using different per-gene and per-module methods of network inference.** A. Performance measured using Area under the precision recall (AUPR) curve. B. Performance measured using fold enrichment of predicted network edges to true network edges, where fold enrichment is defined as the ratio of the observed and expected fraction of true edges.
(PDF)

Figure S2 **Comparison of MERLIN to per-gene linear regression approach based on prediction error.** Shown are the number of genes in which MERLIN is significantly better or worse than the linear regression-based per-gene approach. Prediction of expression is evaluated using Pearson's correlation between true and predicted expression of a gene using five-fold cross validation. The Pearson's correlation in the five folds are used to test whether the correlations are significantly higher or lower between two methods using a one-sided t-test.
(PDF)

Figure S3
**Effect of MERLIN hyper-parameter values on network reconstruction performance for high modularity networks.** Shown are the F-scores for networks of different sizes of high modularity for different parameter settings of sparsity (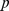), module effect (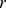) and clustering threshold (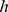).(PDF)

Figure S4
**Effect of MERLIN hyper-parameter values on network reconstruction performance for high modularity networks.** Shown are the F-scores for networks of different sizes of low modularity for different parameter settings of sparsity (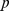), module effect (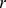) and clustering threshold (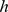).(PDF)

Figure S5
**Effect of different parameters on network structure recovery and module level statistics.** Shown are the F-scores on two sets of three networks with 100, 300 and 500 nodes, the top set has high modularity and the bottom set has low modularity. The modularity during network generation was controlled by a parameter specifying the probability with which a target and regulator come from the same module. F-score and module statistics are shown for different values of parameter settings controlling sparsity (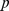), modularity (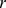) and the height of the tree that determines modules during hierarchical clustering (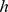). Two module level statistics are shown: number of modules (red-white scale, upper diagonal); fraction of genes included in good-sized modules (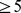 genes, lower white-black triangle.(PDF)

Figure S6
**Effect of module effect hyper-parameter on regulatory modularity of the inferred network.** Shown are measured regulatory modularity of the inferred network for different values of the module effect parameter (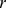). Shown are the estimated modularities in networks of different sizes (different colors), at 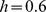 and 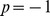. for a network which has a high probability (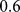) of regulators and targets to come from the same module (left), and for a network which has lower probability of regulators and targets to come from the same module (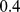).(PDF)

Table S1
**Gene Ontology process enrichment for MERLIN modules on yeast stress data.** This table lists the Gene Ontology process enrichment for the modules/clusters inferred using MERLIN on Gasch stress data. Each row corresponds to a GO process and the columns correspond to the Module ID, term name, 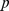-value, FDR, Total genes with annotation, Number of genes annotated with the term, Size of the module/cluster, Number of genes annotated with the term in the cluster, fold enrichment of the term in the cluster, the genes contributing to the term enrichment.(TXT)

Table S2 **Gene Ontology process enrichment for MERLIN modules inferred on human ES cell differentiation data.** This table lists the Gene Ontology process enrichment for the modules inferred using MERLIN on the human ES cell differentiation data. The file format is as in **Table S1**.
(TXT)

Table S3 **MSigDB term enrichment for MERLIN modules inferred on human ES cell differentiation data.** This table lists the MSigDB term enrichment for the modules inferred using MERLIN on the human ES cell differentiation data. The file format is as in **Table S1**.
(TXT)

Text S1 **Pseudo code and additional details of the MERLIN algorithm.**
(PDF)
