# Supplementary figures and images for: Integrated Module and Gene-Specific Regulatory Inference Implicates Upstream Signaling Networks
Source: PLoS Comput Biol. 2013 Oct 17;9(10):e1003252. doi: 10.1371/journal.pcbi.1003252 (PMC3798279; doi:10.1371/journal.pcbi.1003252)

Figure S1

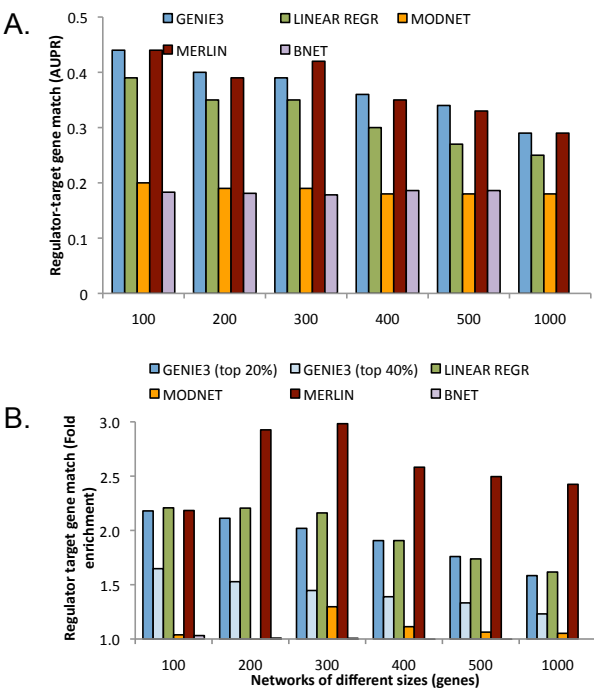

Supplement: Figure S1 — Edge-based comparisons on simulated networks of different sizes using different per-gene and per-module methods of network inference. A. Performance measured using Area under the precision recall (AUPR) curve. B. Performance measured using fold enrichment of predicted network edges to true network edges, where fold enrichment is defined as the ratio of the observed and expected fraction of true edges. (PDF) [file pcbi.1003252.s001.pdf]

**Figure S2**

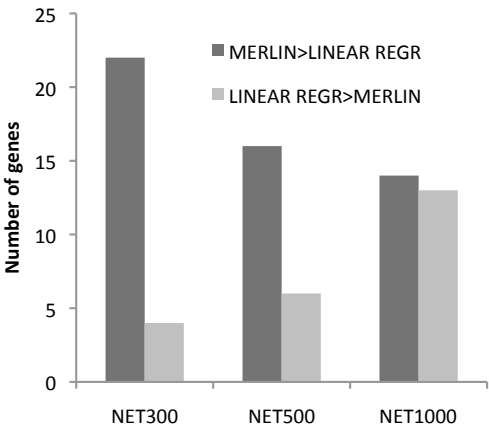

Supplement: Figure S2 — Comparison of MERLIN to per-gene linear regression approach based on prediction error. Shown are the number of genes in which MERLIN is significantly better or worse than the linear regression-based per-gene approach. Prediction of expression is evaluated using Pearson's correlation between true and predicted expression of a gene using five-fold cross validation. The Pearson's correlation in the five folds are used to test whether the correlations are significantly higher or lower between two methods using a one-sided t-test. (PDF) [file pcbi.1003252.s002.pdf]

Figure S5

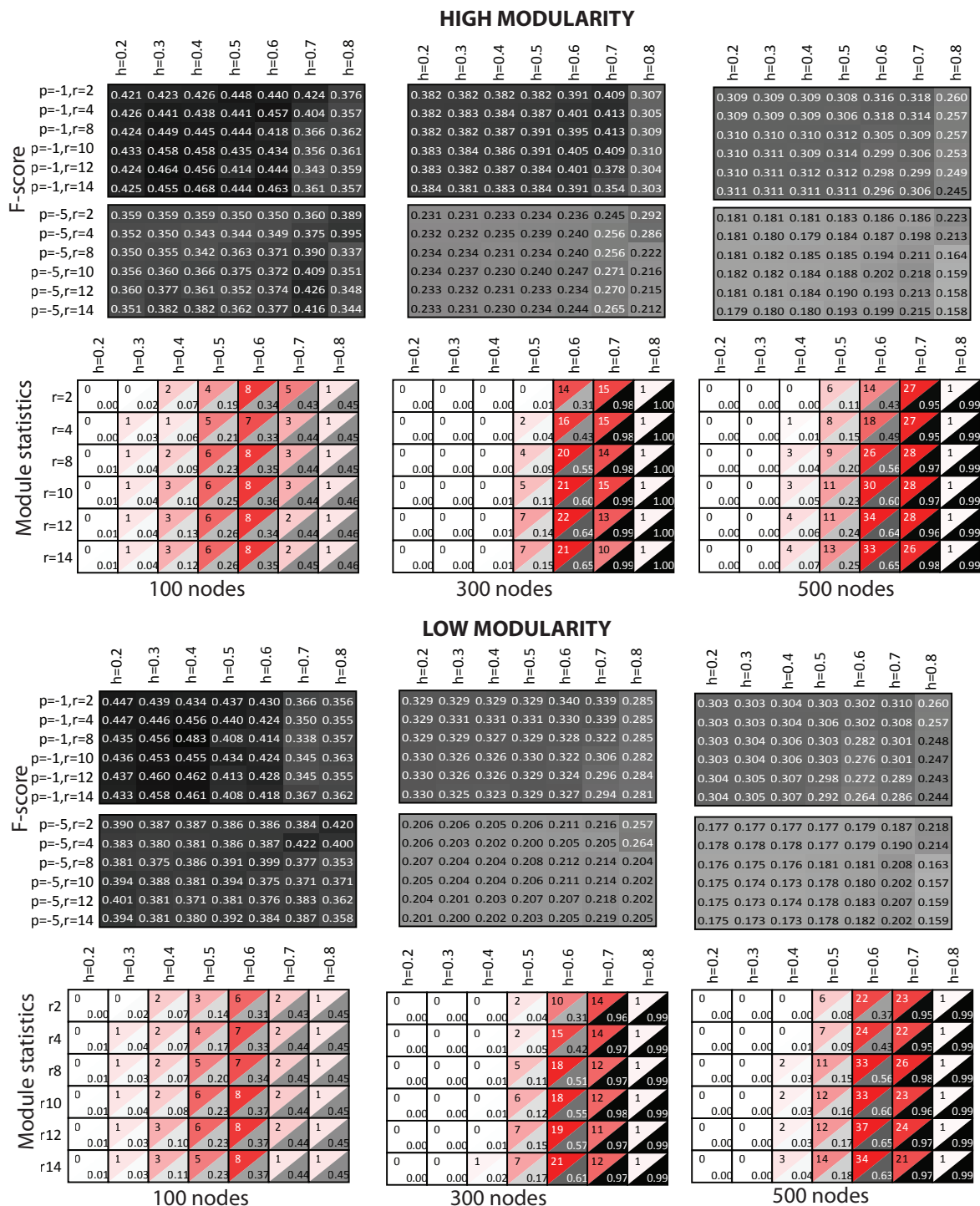

Supplement: Figure S5 — Effect of different parameters on network structure recovery and module level statistics. Shown are the F-scores on two sets of three networks with 100, 300 and 500 nodes, the top set has high modularity and the bottom set has low modularity. The modularity during network generation was controlled by a parameter specifying the probability with which a target and regulator come from the same module. F-score and module statistics are shown for different values of parameter settings controlling sparsity (), modularity () and the height of the tree that determines modules during hierarchical clustering (). Two module level statistics are shown: number of modules (red-white scale, upper diagonal); fraction of genes included in good-sized modules ( genes, lower white-black triangle. (PDF) [file pcbi.1003252.s005.pdf]

Figure S6

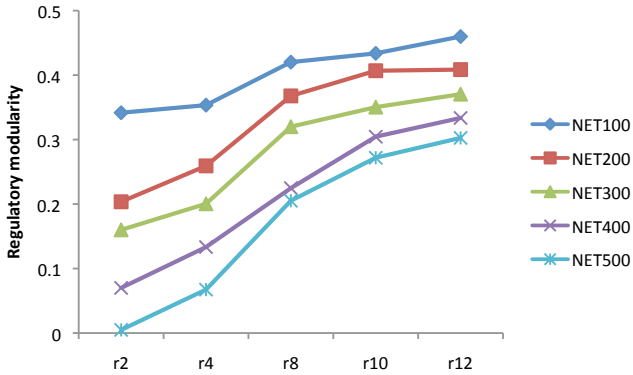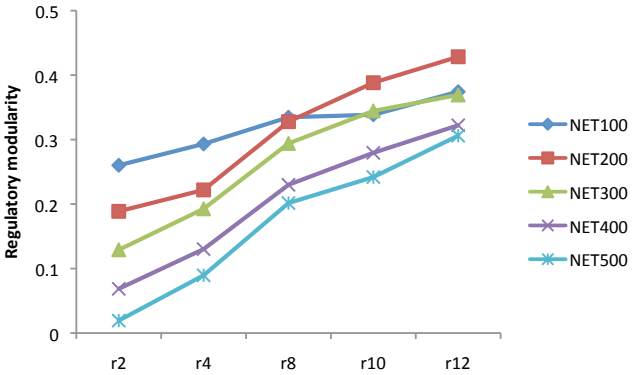

Supplement: Figure S6 — Effect of module effect hyper-parameter on regulatory modularity of the inferred network. Shown are measured regulatory modularity of the inferred network for different values of the module effect parameter (). Shown are the estimated modularities in networks of different sizes (different colors), at and . for a network which has a high probability () of regulators and targets to come from the same module (left), and for a network which has lower probability of regulators and targets to come from the same module (). (PDF) [file pcbi.1003252.s006.pdf]
