# Supplementary material for: Integrated Module and Gene-Specific Regulatory Inference Implicates Upstream Signaling Networks
Source: PLoS Comput Biol. 2013 Oct 17;9(10):e1003252. doi: 10.1371/journal.pcbi.1003252 (PMC3798279; doi:10.1371/journal.pcbi.1003252)
Supplement: Figure S3 — Effect of MERLIN hyper-parameter values on network reconstruction performance for high modularity networks. Shown are the F-scores for networks of different sizes of high modularity for different parameter settings of sparsity (), module effect () and clustering threshold (). (PDF) [file pcbi.1003252.s003.pdf]

# Figure S3

## HIGH MODULARITY NETWORKS

| | p=-1 | p=-5 | p=-10 | p=-15 | p=-20 |
|---|---|---|---|---|---|
| r=2_h=0.2 | 0.421 | 0.359 | 0.210 | 0.141 | 0.069 |
| r=2_h=0.3 | 0.423 | 0.359 | 0.210 | 0.141 | 0.069 |
| r=2_h=0.4 | 0.426 | 0.359 | 0.210 | 0.141 | 0.069 |
| r=2_h=0.5 | 0.448 | 0.350 | 0.210 | 0.141 | 0.069 |
| r=2_h=0.6 | 0.440 | 0.350 | 0.210 | 0.141 | 0.069 |
| r=2_h=0.7 | 0.424 | 0.360 | 0.210 | 0.141 | 0.069 |
| r=2_h=0.8 | 0.376 | 0.389 | 0.216 | 0.149 | 0.069 |
| r=4_h=0.2 | 0.426 | 0.352 | 0.202 | 0.150 | 0.078 |
| r=4_h=0.3 | 0.441 | 0.350 | 0.193 | 0.150 | 0.078 |
| r=4_h=0.4 | 0.438 | 0.343 | 0.201 | 0.150 | 0.078 |
| r=4_h=0.5 | 0.441 | 0.344 | 0.209 | 0.150 | 0.078 |
| r=4_h=0.6 | 0.457 | 0.349 | 0.209 | 0.150 | 0.078 |
| r=4_h=0.7 | 0.404 | 0.375 | 0.216 | 0.150 | 0.078 |
| r=4_h=0.8 | 0.357 | 0.395 | 0.235 | 0.140 | 0.078 |
| r=8_h=0.2 | 0.424 | 0.350 | 0.209 | 0.158 | 0.068 |
| r=8_h=0.3 | 0.449 | 0.355 | 0.223 | 0.158 | 0.068 |
| r=8_h=0.4 | 0.445 | 0.342 | 0.223 | 0.157 | 0.068 |
| r=8_h=0.5 | 0.444 | 0.363 | 0.231 | 0.157 | 0.068 |
| r=8_h=0.6 | 0.418 | 0.371 | 0.230 | 0.157 | 0.068 |
| r=8_h=0.7 | 0.366 | 0.390 | 0.225 | 0.161 | 0.068 |
| r=8_h=0.8 | 0.362 | 0.337 | 0.260 | 0.162 | 0.078 |
| r=10_h=0.2 | 0.433 | 0.356 | 0.207 | 0.158 | 0.068 |
| r=10_h=0.3 | 0.458 | 0.360 | 0.214 | 0.158 | 0.068 |
| r=10_h=0.4 | 0.458 | 0.366 | 0.214 | 0.157 | 0.068 |
| r=10_h=0.5 | 0.435 | 0.375 | 0.221 | 0.157 | 0.068 |
| r=10_h=0.6 | 0.434 | 0.372 | 0.228 | 0.157 | 0.068 |
| r=10_h=0.7 | 0.356 | 0.409 | 0.219 | 0.170 | 0.068 |
| r=10_h=0.8 | 0.361 | 0.351 | 0.255 | 0.178 | 0.087 |
| r=12_h=0.2 | 0.424 | 0.360 | 0.230 | 0.158 | 0.068 |
| r=12_h=0.3 | 0.464 | 0.377 | 0.229 | 0.158 | 0.068 |
| r=12_h=0.4 | 0.456 | 0.361 | 0.229 | 0.157 | 0.068 |
| r=12_h=0.5 | 0.414 | 0.352 | 0.229 | 0.157 | 0.068 |
| r=12_h=0.6 | 0.444 | 0.374 | 0.235 | 0.157 | 0.068 |
| r=12_h=0.7 | 0.343 | 0.426 | 0.235 | 0.170 | 0.067 |
| r=12_h=0.8 | 0.359 | 0.348 | 0.256 | 0.181 | 0.087 |
| r=14_h=0.2 | 0.425 | 0.351 | 0.230 | 0.157 | 0.068 |
| r=14_h=0.3 | 0.455 | 0.382 | 0.259 | 0.157 | 0.068 |
| r=14_h=0.4 | 0.468 | 0.382 | 0.257 | 0.157 | 0.068 |
| r=14_h=0.5 | 0.444 | 0.362 | 0.250 | 0.157 | 0.068 |
| r=14_h=0.6 | 0.463 | 0.377 | 0.264 | 0.157 | 0.068 |
| r=14_h=0.7 | 0.361 | 0.416 | 0.247 | 0.168 | 0.067 |
| r=14_h=0.8 | 0.357 | 0.344 | 0.229 | 0.165 | 0.086 |

Net100

| | p=-1 | p=-5 | p=-10 | p=-15 | p=-20 |
|---|---|---|---|---|---|
| r=2_h=0.2 | 0.366 | 0.281 | 0.163 | 0.101 | 0.068 |
| r=2_h=0.3 | 0.366 | 0.277 | 0.163 | 0.101 | 0.068 |
| r=2_h=0.4 | 0.367 | 0.279 | 0.163 | 0.101 | 0.068 |
| r=2_h=0.5 | 0.370 | 0.276 | 0.165 | 0.101 | 0.068 |
| r=2_h=0.6 | 0.382 | 0.279 | 0.167 | 0.104 | 0.068 |
| r=2_h=0.7 | 0.376 | 0.283 | 0.173 | 0.104 | 0.070 |
| r=2_h=0.8 | 0.322 | 0.320 | 0.194 | 0.115 | 0.068 |
| r=4_h=0.2 | 0.369 | 0.285 | 0.163 | 0.104 | 0.073 |
| r=4_h=0.3 | 0.370 | 0.281 | 0.163 | 0.104 | 0.073 |
| r=4_h=0.4 | 0.371 | 0.286 | 0.165 | 0.104 | 0.073 |
| r=4_h=0.5 | 0.385 | 0.289 | 0.165 | 0.104 | 0.073 |
| r=4_h=0.6 | 0.390 | 0.283 | 0.169 | 0.113 | 0.073 |
| r=4_h=0.7 | 0.360 | 0.304 | 0.175 | 0.115 | 0.073 |
| r=4_h=0.8 | 0.317 | 0.324 | 0.229 | 0.141 | 0.077 |
| r=8_h=0.2 | 0.370 | 0.287 | 0.166 | 0.113 | 0.070 |
| r=8_h=0.3 | 0.372 | 0.284 | 0.164 | 0.113 | 0.070 |
| r=8_h=0.4 | 0.379 | 0.291 | 0.166 | 0.111 | 0.070 |
| r=8_h=0.5 | 0.392 | 0.285 | 0.177 | 0.106 | 0.070 |
| r=8_h=0.6 | 0.373 | 0.289 | 0.176 | 0.117 | 0.073 |
| r=8_h=0.7 | 0.312 | 0.296 | 0.198 | 0.134 | 0.086 |
| r=8_h=0.8 | 0.322 | 0.248 | 0.254 | 0.152 | 0.088 |
| r=10_h=0.2 | 0.371 | 0.285 | 0.162 | 0.113 | 0.070 |
| r=10_h=0.3 | 0.376 | 0.284 | 0.162 | 0.115 | 0.070 |
| r=10_h=0.4 | 0.382 | 0.291 | 0.160 | 0.113 | 0.070 |
| r=10_h=0.5 | 0.380 | 0.290 | 0.164 | 0.108 | 0.070 |
| r=10_h=0.6 | 0.377 | 0.301 | 0.174 | 0.116 | 0.073 |
| r=10_h=0.7 | 0.313 | 0.290 | 0.196 | 0.140 | 0.093 |
| r=10_h=0.8 | 0.318 | 0.257 | 0.223 | 0.166 | 0.101 |
| r=12_h=0.2 | 0.370 | 0.283 | 0.167 | 0.108 | 0.070 |
| r=12_h=0.3 | 0.375 | 0.281 | 0.165 | 0.111 | 0.070 |
| r=12_h=0.4 | 0.381 | 0.288 | 0.163 | 0.108 | 0.070 |
| r=12_h=0.5 | 0.375 | 0.293 | 0.166 | 0.106 | 0.070 |
| r=12_h=0.6 | 0.367 | 0.293 | 0.180 | 0.114 | 0.075 |
| r=12_h=0.7 | 0.302 | 0.303 | 0.215 | 0.133 | 0.095 |
| r=12_h=0.8 | 0.317 | 0.257 | 0.196 | 0.186 | 0.108 |
| r=14_h=0.2 | 0.367 | 0.284 | 0.169 | 0.101 | 0.073 |
| r=14_h=0.3 | 0.374 | 0.283 | 0.169 | 0.104 | 0.073 |
| r=14_h=0.4 | 0.378 | 0.286 | 0.171 | 0.104 | 0.073 |
| r=14_h=0.5 | 0.377 | 0.289 | 0.176 | 0.104 | 0.073 |
| r=14_h=0.6 | 0.368 | 0.290 | 0.180 | 0.110 | 0.075 |
| r=14_h=0.7 | 0.298 | 0.316 | 0.215 | 0.138 | 0.097 |
| r=14_h=0.8 | 0.318 | 0.260 | 0.188 | 0.183 | 0.108 |

Net200

| | p=-1 | p=-5 | p=-10 | p=-15 | p=-20 |
|---|---|---|---|---|---|
| r=2_h=0.2 | 0.382 | 0.231 | 0.110 | 0.053 | 0.032 |
| r=2_h=0.3 | 0.382 | 0.231 | 0.112 | 0.053 | 0.032 |
| r=2_h=0.4 | 0.382 | 0.233 | 0.112 | 0.054 | 0.032 |
| r=2_h=0.5 | 0.382 | 0.234 | 0.111 | 0.054 | 0.032 |
| r=2_h=0.6 | 0.391 | 0.236 | 0.111 | 0.055 | 0.032 |
| r=2_h=0.7 | 0.409 | 0.245 | 0.119 | 0.055 | 0.033 |
| r=2_h=0.8 | 0.307 | 0.292 | 0.145 | 0.057 | 0.033 |
| r=4_h=0.2 | 0.382 | 0.232 | 0.113 | 0.055 | 0.033 |
| r=4_h=0.3 | 0.383 | 0.232 | 0.113 | 0.056 | 0.033 |
| r=4_h=0.4 | 0.384 | 0.235 | 0.111 | 0.056 | 0.032 |
| r=4_h=0.5 | 0.387 | 0.239 | 0.111 | 0.055 | 0.032 |
| r=4_h=0.6 | 0.401 | 0.240 | 0.113 | 0.056 | 0.032 |
| r=4_h=0.7 | 0.413 | 0.256 | 0.125 | 0.059 | 0.035 |
| r=4_h=0.8 | 0.305 | 0.286 | 0.161 | 0.073 | 0.035 |
| r=8_h=0.2 | 0.382 | 0.234 | 0.113 | 0.053 | 0.031 |
| r=8_h=0.3 | 0.382 | 0.234 | 0.114 | 0.054 | 0.031 |
| r=8_h=0.4 | 0.387 | 0.231 | 0.113 | 0.054 | 0.031 |
| r=8_h=0.5 | 0.391 | 0.234 | 0.113 | 0.054 | 0.031 |
| r=8_h=0.6 | 0.395 | 0.240 | 0.116 | 0.054 | 0.031 |
| r=8_h=0.7 | 0.413 | 0.256 | 0.132 | 0.063 | 0.037 |
| r=8_h=0.8 | 0.309 | 0.222 | 0.198 | 0.085 | 0.041 |
| r=10_h=0.2 | 0.383 | 0.234 | 0.111 | 0.056 | 0.031 |
| r=10_h=0.3 | 0.384 | 0.237 | 0.112 | 0.057 | 0.031 |
| r=10_h=0.4 | 0.386 | 0.230 | 0.114 | 0.057 | 0.031 |
| r=10_h=0.5 | 0.391 | 0.240 | 0.114 | 0.056 | 0.030 |
| r=10_h=0.6 | 0.405 | 0.247 | 0.116 | 0.056 | 0.030 |
| r=10_h=0.7 | 0.409 | 0.271 | 0.128 | 0.073 | 0.038 |
| r=10_h=0.8 | 0.310 | 0.216 | 0.174 | 0.095 | 0.046 |
| r=12_h=0.2 | 0.383 | 0.233 | 0.111 | 0.055 | 0.033 |
| r=12_h=0.3 | 0.382 | 0.232 | 0.113 | 0.054 | 0.033 |
| r=12_h=0.4 | 0.387 | 0.231 | 0.115 | 0.054 | 0.033 |
| r=12_h=0.5 | 0.384 | 0.233 | 0.117 | 0.053 | 0.031 |
| r=12_h=0.6 | 0.401 | 0.234 | 0.117 | 0.053 | 0.031 |
| r=12_h=0.7 | 0.378 | 0.270 | 0.132 | 0.070 | 0.042 |
| r=12_h=0.8 | 0.304 | 0.215 | 0.149 | 0.108 | 0.047 |
| r=14_h=0.2 | 0.384 | 0.233 | 0.110 | 0.055 | 0.035 |
| r=14_h=0.3 | 0.381 | 0.231 | 0.112 | 0.055 | 0.035 |
| r=14_h=0.4 | 0.383 | 0.230 | 0.116 | 0.055 | 0.035 |
| r=14_h=0.5 | 0.384 | 0.234 | 0.115 | 0.054 | 0.032 |
| r=14_h=0.6 | 0.391 | 0.244 | 0.117 | 0.054 | 0.032 |
| r=14_h=0.7 | 0.354 | 0.265 | 0.137 | 0.072 | 0.046 |
| r=14_h=0.8 | 0.303 | 0.212 | 0.142 | 0.120 | 0.049 |

Net300

| | p=-1 | p=-5 | p=-10 | p=-15 | p=-20 |
|---|---|---|---|---|---|
| r=2_h=0 | 0.342 | 0.192 | 0.101 | 0.061 | 0.043 |
| r=2_h=0 | 0.341 | 0.192 | 0.101 | 0.062 | 0.043 |
| r=2_h=0 | 0.342 | 0.192 | 0.101 | 0.062 | 0.043 |
| r=2_h=0 | 0.344 | 0.195 | 0.101 | 0.062 | 0.043 |
| r=2_h=0 | 0.347 | 0.198 | 0.101 | 0.062 | 0.043 |
| r=2_h=0 | 0.345 | 0.201 | 0.104 | 0.063 | 0.044 |
| r=2_h=0 | 0.297 | 0.246 | 0.126 | 0.069 | 0.045 |
| r=4_h=0 | 0.342 | 0.193 | 0.102 | 0.064 | 0.043 |
| r=4_h=0 | 0.342 | 0.192 | 0.103 | 0.064 | 0.043 |
| r=4_h=0 | 0.341 | 0.193 | 0.106 | 0.064 | 0.043 |
| r=4_h=0 | 0.347 | 0.190 | 0.106 | 0.065 | 0.043 |
| r=4_h=0 | 0.350 | 0.194 | 0.106 | 0.065 | 0.043 |
| r=4_h=0 | 0.350 | 0.209 | 0.114 | 0.067 | 0.045 |
| r=4_h=0 | 0.291 | 0.233 | 0.144 | 0.082 | 0.050 |
| r=8_h=0 | 0.342 | 0.193 | 0.104 | 0.067 | 0.044 |
| r=8_h=0 | 0.345 | 0.192 | 0.104 | 0.067 | 0.044 |
| r=8_h=0 | 0.347 | 0.194 | 0.111 | 0.068 | 0.044 |
| r=8_h=0 | 0.351 | 0.195 | 0.114 | 0.068 | 0.044 |
| r=8_h=0 | 0.332 | 0.201 | 0.115 | 0.068 | 0.044 |
| r=8_h=0 | 0.345 | 0.215 | 0.123 | 0.079 | 0.053 |
| r=8_h=0 | 0.285 | 0.185 | 0.169 | 0.093 | 0.064 |
| r=10_h=0 | 0.343 | 0.196 | 0.105 | 0.068 | 0.044 |
| r=10_h=0 | 0.346 | 0.191 | 0.106 | 0.068 | 0.044 |
| r=10_h=0 | 0.346 | 0.197 | 0.111 | 0.069 | 0.044 |
| r=10_h=0 | 0.350 | 0.199 | 0.116 | 0.069 | 0.044 |
| r=10_h=0 | 0.334 | 0.204 | 0.116 | 0.067 | 0.044 |
| r=10_h=0 | 0.322 | 0.211 | 0.128 | 0.082 | 0.054 |
| r=10_h=0 | 0.285 | 0.181 | 0.140 | 0.108 | 0.058 |
| r=12_h=0 | 0.343 | 0.196 | 0.106 | 0.068 | 0.044 |
| r=12_h=0 | 0.349 | 0.189 | 0.106 | 0.068 | 0.043 |
| r=12_h=0 | 0.349 | 0.194 | 0.114 | 0.071 | 0.043 |
| r=12_h=0 | 0.342 | 0.196 | 0.118 | 0.070 | 0.043 |
| r=12_h=0 | 0.325 | 0.201 | 0.118 | 0.071 | 0.043 |
| r=12_h=0 | 0.325 | 0.218 | 0.129 | 0.087 | 0.055 |
| r=12_h=0 | 0.283 | 0.181 | 0.127 | 0.118 | 0.059 |
| r=14_h=0 | 0.343 | 0.195 | 0.105 | 0.068 | 0.045 |
| r=14_h=0 | 0.349 | 0.190 | 0.106 | 0.068 | 0.045 |
| r=14_h=0 | 0.352 | 0.190 | 0.117 | 0.073 | 0.045 |
| r=14_h=0 | 0.342 | 0.200 | 0.119 | 0.073 | 0.045 |
| r=14_h=0 | 0.323 | 0.204 | 0.121 | 0.074 | 0.044 |
| r=14_h=0 | 0.321 | 0.216 | 0.134 | 0.094 | 0.059 |
| r=14_h=0 | 0.280 | 0.181 | 0.124 | 0.104 | 0.066 |

Net400

| | p=-1 | p=-5 | p=-10 | p=-15 | p=-20 |
|---|---|---|---|---|---|
| r=2_h=0.2 | 0.309 | 0.181 | 0.106 | 0.071 | 0.050 |
| r=2_h=0.3 | 0.309 | 0.181 | 0.106 | 0.071 | 0.050 |
| r=2_h=0.4 | 0.309 | 0.181 | 0.106 | 0.071 | 0.050 |
| r=2_h=0.5 | 0.308 | 0.183 | 0.106 | 0.071 | 0.050 |
| r=2_h=0.6 | 0.316 | 0.186 | 0.106 | 0.071 | 0.051 |
| r=2_h=0.7 | 0.318 | 0.186 | 0.111 | 0.074 | 0.051 |
| r=2_h=0.8 | 0.260 | 0.223 | 0.129 | 0.078 | 0.053 |
| r=4_h=0.2 | 0.309 | 0.181 | 0.106 | 0.070 | 0.051 |
| r=4_h=0.3 | 0.309 | 0.180 | 0.107 | 0.070 | 0.051 |
| r=4_h=0.4 | 0.309 | 0.179 | 0.107 | 0.071 | 0.051 |
| r=4_h=0.5 | 0.306 | 0.184 | 0.108 | 0.071 | 0.051 |
| r=4_h=0.6 | 0.318 | 0.187 | 0.107 | 0.071 | 0.051 |
| r=4_h=0.7 | 0.314 | 0.198 | 0.115 | 0.076 | 0.053 |
| r=4_h=0.8 | 0.257 | 0.213 | 0.145 | 0.091 | 0.058 |
| r=8_h=0.2 | 0.310 | 0.181 | 0.105 | 0.071 | 0.053 |
| r=8_h=0.3 | 0.310 | 0.182 | 0.109 | 0.074 | 0.053 |
| r=8_h=0.4 | 0.310 | 0.185 | 0.108 | 0.076 | 0.053 |
| r=8_h=0.5 | 0.312 | 0.185 | 0.110 | 0.078 | 0.053 |
| r=8_h=0.6 | 0.305 | 0.194 | 0.116 | 0.078 | 0.054 |
| r=8_h=0.7 | 0.309 | 0.211 | 0.123 | 0.083 | 0.058 |
| r=8_h=0.8 | 0.257 | 0.164 | 0.160 | 0.107 | 0.070 |
| r=10_h=0.2 | 0.310 | 0.182 | 0.106 | 0.072 | 0.054 |
| r=10_h=0.3 | 0.311 | 0.182 | 0.109 | 0.072 | 0.054 |
| r=10_h=0.4 | 0.309 | 0.184 | 0.107 | 0.077 | 0.054 |
| r=10_h=0.5 | 0.314 | 0.188 | 0.111 | 0.078 | 0.054 |
| r=10_h=0.6 | 0.299 | 0.202 | 0.111 | 0.079 | 0.056 |
| r=10_h=0.7 | 0.306 | 0.218 | 0.122 | 0.087 | 0.064 |
| r=10_h=0.8 | 0.253 | 0.159 | 0.140 | 0.119 | 0.070 |
| r=12_h=0.2 | 0.310 | 0.181 | 0.106 | 0.072 | 0.054 |
| r=12_h=0.3 | 0.311 | 0.181 | 0.107 | 0.075 | 0.056 |
| r=12_h=0.4 | 0.312 | 0.184 | 0.109 | 0.078 | 0.056 |
| r=12_h=0.5 | 0.312 | 0.190 | 0.112 | 0.078 | 0.056 |
| r=12_h=0.6 | 0.298 | 0.193 | 0.117 | 0.080 | 0.057 |
| r=12_h=0.7 | 0.299 | 0.213 | 0.126 | 0.091 | 0.066 |
| r=12_h=0.8 | 0.249 | 0.158 | 0.124 | 0.128 | 0.078 |
| r=14_h=0.2 | 0.311 | 0.179 | 0.107 | 0.073 | 0.054 |
| r=14_h=0.3 | 0.311 | 0.180 | 0.108 | 0.075 | 0.056 |
| r=14_h=0.4 | 0.311 | 0.180 | 0.109 | 0.077 | 0.056 |
| r=14_h=0.5 | 0.311 | 0.193 | 0.114 | 0.079 | 0.056 |
| r=14_h=0.6 | 0.296 | 0.199 | 0.116 | 0.080 | 0.058 |
| r=14_h=0.7 | 0.306 | 0.215 | 0.128 | 0.090 | 0.069 |
| r=14_h=0.8 | 0.245 | 0.158 | 0.117 | 0.109 | 0.086 |

Net500
