# Supplementary material for: Integrated Module and Gene-Specific Regulatory Inference Implicates Upstream Signaling Networks
Source: PLoS Comput Biol. 2013 Oct 17;9(10):e1003252. doi: 10.1371/journal.pcbi.1003252 (PMC3798279; doi:10.1371/journal.pcbi.1003252)
Supplement: Figure S4 — Effect of MERLIN hyper-parameter values on network reconstruction performance for high modularity networks. Shown are the F-scores for networks of different sizes of low modularity for different parameter settings of sparsity (), module effect () and clustering threshold (). (PDF) [file pcbi.1003252.s004.pdf]

Figure S4

LOW MODULARITY

Net100

	p=-1	p=-5	p=-10	p=-15	p=-20
r=2_h=0.2	0.447	0.390	0.254	0.191	0.150
r=2_h=0.3	0.439	0.387	0.254	0.191	0.150
r=2_h=0.4	0.434	0.387	0.254	0.191	0.150
r=2_h=0.5	0.437	0.386	0.254	0.191	0.150
r=2_h=0.6	0.430	0.386	0.262	0.191	0.150
r=2_h=0.7	0.366	0.384	0.280	0.191	0.150
r=2_h=0.8	0.356	0.420	0.291	0.190	0.150
r=4_h=0.2	0.447	0.383	0.254	0.190	0.150
r=4_h=0.3	0.446	0.380	0.254	0.190	0.150
r=4_h=0.4	0.456	0.381	0.254	0.190	0.150
r=4_h=0.5	0.440	0.386	0.254	0.190	0.150
r=4_h=0.6	0.424	0.387	0.276	0.190	0.158
r=4_h=0.7	0.350	0.422	0.270	0.197	0.158
r=4_h=0.8	0.355	0.400	0.309	0.187	0.158
r=8_h=0.2	0.435	0.381	0.263	0.190	0.150
r=8_h=0.3	0.456	0.375	0.263	0.190	0.150
r=8_h=0.4	0.483	0.386	0.263	0.190	0.150
r=8_h=0.5	0.408	0.391	0.270	0.190	0.150
r=8_h=0.6	0.414	0.399	0.290	0.205	0.167
r=8_h=0.7	0.338	0.377	0.316	0.201	0.164
r=8_h=0.8	0.357	0.353	0.348	0.214	0.163
r=10_h=0.2	0.436	0.394	0.263	0.189	0.150
r=10_h=0.3	0.453	0.388	0.263	0.189	0.150
r=10_h=0.4	0.455	0.381	0.263	0.189	0.150
r=10_h=0.5	0.434	0.394	0.270	0.189	0.150
r=10_h=0.6	0.424	0.375	0.290	0.204	0.174
r=10_h=0.7	0.345	0.371	0.305	0.197	0.176
r=10_h=0.8	0.363	0.371	0.340	0.225	0.171
r=12_h=0.2	0.437	0.401	0.263	0.181	0.150
r=12_h=0.3	0.460	0.381	0.263	0.181	0.150
r=12_h=0.4	0.462	0.371	0.263	0.181	0.150
r=12_h=0.5	0.413	0.381	0.270	0.181	0.150
r=12_h=0.6	0.428	0.376	0.283	0.204	0.182
r=12_h=0.7	0.345	0.383	0.300	0.197	0.172
r=12_h=0.8	0.355	0.362	0.302	0.218	0.171
r=14_h=0.2	0.433	0.394	0.270	0.181	0.150
r=14_h=0.3	0.458	0.381	0.270	0.180	0.150
r=14_h=0.4	0.461	0.380	0.263	0.180	0.150
r=14_h=0.5	0.408	0.392	0.270	0.180	0.150
r=14_h=0.6	0.418	0.384	0.283	0.195	0.182
r=14_h=0.7	0.367	0.387	0.295	0.189	0.178
r=14_h=0.8	0.362	0.358	0.287	0.228	0.173

Net200

	p=-1	p=-5	p=-10	p=-15	p=-20
r=2_h=0.2	0.352	0.251	0.141	0.095	0.063
r=2_h=0.3	0.352	0.249	0.143	0.095	0.063
r=2_h=0.4	0.354	0.251	0.143	0.095	0.063
r=2_h=0.5	0.366	0.255	0.143	0.095	0.063
r=2_h=0.6	0.372	0.254	0.143	0.095	0.063
r=2_h=0.7	0.365	0.266	0.145	0.099	0.061
r=2_h=0.8	0.302	0.312	0.173	0.106	0.061
r=4_h=0.2	0.351	0.250	0.143	0.095	0.065
r=4_h=0.3	0.352	0.250	0.146	0.092	0.065
r=4_h=0.4	0.360	0.252	0.145	0.095	0.065
r=4_h=0.5	0.368	0.262	0.147	0.095	0.068
r=4_h=0.6	0.372	0.268	0.147	0.095	0.068
r=4_h=0.7	0.335	0.291	0.163	0.105	0.070
r=4_h=0.8	0.304	0.315	0.205	0.119	0.067
r=8_h=0.2	0.353	0.250	0.146	0.099	0.068
r=8_h=0.3	0.355	0.249	0.147	0.099	0.068
r=8_h=0.4	0.361	0.249	0.151	0.099	0.068
r=8_h=0.5	0.370	0.265	0.160	0.099	0.068
r=8_h=0.6	0.367	0.282	0.160	0.099	0.068
r=8_h=0.7	0.307	0.298	0.188	0.126	0.067
r=8_h=0.8	0.301	0.254	0.231	0.139	0.074
r=10_h=0.2	0.353	0.249	0.145	0.101	0.065
r=10_h=0.3	0.359	0.244	0.150	0.101	0.065
r=10_h=0.4	0.369	0.253	0.156	0.101	0.065
r=10_h=0.5	0.366	0.264	0.162	0.103	0.065
r=10_h=0.6	0.367	0.277	0.159	0.103	0.065
r=10_h=0.7	0.294	0.291	0.186	0.120	0.076
r=10_h=0.8	0.304	0.253	0.210	0.147	0.077
r=12_h=0.2	0.352	0.249	0.148	0.101	0.068
r=12_h=0.3	0.361	0.241	0.152	0.101	0.067
r=12_h=0.4	0.371	0.254	0.156	0.101	0.068
r=12_h=0.5	0.371	0.274	0.161	0.103	0.068
r=12_h=0.6	0.369	0.280	0.161	0.103	0.068
r=12_h=0.7	0.295	0.278	0.183	0.118	0.081
r=12_h=0.8	0.298	0.252	0.186	0.178	0.086
r=14_h=0.2	0.353	0.245	0.148	0.099	0.068
r=14_h=0.3	0.359	0.240	0.152	0.099	0.070
r=14_h=0.4	0.364	0.260	0.149	0.099	0.068
r=14_h=0.5	0.368	0.280	0.158	0.106	0.068
r=14_h=0.6	0.367	0.276	0.158	0.106	0.068
r=14_h=0.7	0.294	0.304	0.193	0.124	0.080
r=14_h=0.8	0.306	0.252	0.176	0.157	0.094

Net300

	p=-1	p=-5	p=-10	p=-15	p=-20
r=2_h=0.2	0.329	0.206	0.113	0.074	0.037
r=2_h=0.3	0.329	0.206	0.113	0.072	0.038
r=2_h=0.4	0.329	0.205	0.114	0.072	0.038
r=2_h=0.5	0.329	0.206	0.112	0.072	0.038
r=2_h=0.6	0.340	0.211	0.112	0.072	0.038
r=2_h=0.7	0.339	0.216	0.113	0.072	0.042
r=2_h=0.8	0.285	0.257	0.128	0.076	0.043
r=4_h=0.2	0.329	0.206	0.117	0.073	0.038
r=4_h=0.3	0.331	0.203	0.115	0.072	0.040
r=4_h=0.4	0.331	0.202	0.116	0.073	0.040
r=4_h=0.5	0.331	0.200	0.116	0.073	0.040
r=4_h=0.6	0.330	0.205	0.119	0.073	0.040
r=4_h=0.7	0.339	0.205	0.122	0.072	0.042
r=4_h=0.8	0.285	0.264	0.143	0.082	0.044
r=8_h=0.2	0.329	0.207	0.116	0.074	0.042
r=8_h=0.3	0.329	0.204	0.118	0.074	0.043
r=8_h=0.4	0.327	0.204	0.117	0.075	0.043
r=8_h=0.5	0.329	0.208	0.118	0.075	0.043
r=8_h=0.6	0.328	0.212	0.120	0.075	0.043
r=8_h=0.7	0.322	0.214	0.126	0.088	0.045
r=8_h=0.8	0.285	0.204	0.184	0.101	0.054
r=10_h=0.2	0.330	0.205	0.113	0.076	0.043
r=10_h=0.3	0.326	0.204	0.115	0.076	0.044
r=10_h=0.4	0.326	0.204	0.115	0.075	0.044
r=10_h=0.5	0.330	0.206	0.118	0.076	0.044
r=10_h=0.6	0.322	0.211	0.119	0.078	0.045
r=10_h=0.7	0.306	0.214	0.129	0.084	0.048
r=10_h=0.8	0.282	0.202	0.154	0.121	0.055
r=12_h=0.2	0.330	0.204	0.118	0.076	0.045
r=12_h=0.3	0.326	0.201	0.117	0.075	0.045
r=12_h=0.4	0.326	0.203	0.114	0.074	0.045
r=12_h=0.5	0.329	0.207	0.114	0.075	0.045
r=12_h=0.6	0.324	0.207	0.116	0.078	0.046
r=12_h=0.7	0.296	0.218	0.128	0.089	0.052
r=12_h=0.8	0.284	0.202	0.141	0.142	0.069
r=14_h=0.2	0.330	0.201	0.115	0.077	0.047
r=14_h=0.3	0.325	0.200	0.116	0.077	0.047
r=14_h=0.4	0.323	0.202	0.113	0.076	0.047
r=14_h=0.5	0.329	0.203	0.114	0.076	0.047
r=14_h=0.6	0.327	0.205	0.116	0.079	0.047
r=14_h=0.7	0.294	0.219	0.133	0.091	0.055
r=14_h=0.8	0.281	0.205	0.134	0.138	0.074

Net400

	p=-1	p=-5	p=-10	p=-15	p=-20
r=2_h=0.2	0.298	0.169	0.093	0.058	0.033
r=2_h=0.3	0.298	0.169	0.093	0.058	0.032
r=2_h=0.4	0.298	0.169	0.093	0.058	0.032
r=2_h=0.5	0.297	0.170	0.093	0.058	0.032
r=2_h=0.6	0.302	0.171	0.093	0.058	0.032
r=2_h=0.7	0.299	0.174	0.093	0.060	0.032
r=2_h=0.8	0.256	0.206	0.112	0.063	0.033
r=4_h=0.2	0.298	0.168	0.093	0.060	0.034
r=4_h=0.3	0.297	0.168	0.093	0.060	0.034
r=4_h=0.4	0.297	0.168	0.093	0.060	0.034
r=4_h=0.5	0.298	0.170	0.094	0.060	0.034
r=4_h=0.6	0.290	0.171	0.095	0.060	0.034
r=4_h=0.7	0.299	0.176	0.101	0.062	0.034
r=4_h=0.8	0.254	0.199	0.128	0.074	0.037
r=8_h=0.2	0.298	0.168	0.093	0.060	0.035
r=8_h=0.3	0.298	0.168	0.093	0.061	0.035
r=8_h=0.4	0.299	0.169	0.094	0.061	0.035
r=8_h=0.5	0.293	0.169	0.094	0.061	0.035
r=8_h=0.6	0.288	0.167	0.096	0.061	0.035
r=8_h=0.7	0.282	0.176	0.104	0.068	0.037
r=8_h=0.8	0.252	0.162	0.161	0.082	0.045
r=10_h=0.2	0.298	0.167	0.094	0.060	0.035
r=10_h=0.3	0.297	0.167	0.094	0.060	0.035
r=10_h=0.4	0.297	0.170	0.095	0.060	0.035
r=10_h=0.5	0.296	0.170	0.096	0.060	0.035
r=10_h=0.6	0.284	0.172	0.097	0.060	0.035
r=10_h=0.7	0.278	0.175	0.104	0.069	0.043
r=10_h=0.8	0.249	0.161	0.129	0.098	0.051
r=12_h=0.2	0.298	0.168	0.096	0.060	0.036
r=12_h=0.3	0.296	0.167	0.097	0.061	0.036
r=12_h=0.4	0.295	0.169	0.099	0.061	0.036
r=12_h=0.5	0.293	0.170	0.098	0.061	0.036
r=12_h=0.6	0.273	0.169	0.101	0.061	0.036
r=12_h=0.7	0.282	0.175	0.111	0.072	0.046
r=12_h=0.8	0.250	0.160	0.118	0.120	0.053
r=14_h=0.2	0.298	0.167	0.096	0.061	0.037
r=14_h=0.3	0.297	0.167	0.098	0.061	0.037
r=14_h=0.4	0.294	0.170	0.097	0.061	0.037
r=14_h=0.5	0.286	0.169	0.098	0.061	0.037
r=14_h=0.6	0.269	0.164	0.099	0.061	0.037
r=14_h=0.7	0.278	0.176	0.109	0.068	0.046
r=14_h=0.8	0.244	0.159	0.113	0.115	0.053

Net500

	p=-1	p=-5	p=-10	p=-15	p=-20
r=2_h=0.2	0.303	0.177	0.110	0.080	0.060
r=2_h=0.3	0.303	0.177	0.110	0.080	0.060
r=2_h=0.4	0.304	0.177	0.110	0.080	0.060
r=2_h=0.5	0.303	0.177	0.110	0.080	0.060
r=2_h=0.6	0.302	0.179	0.110	0.080	0.060
r=2_h=0.7	0.310	0.187	0.112	0.080	0.061
r=2_h=0.8	0.260	0.218	0.132	0.088	0.064
r=4_h=0.2	0.303	0.178	0.111	0.081	0.061
r=4_h=0.3	0.303	0.178	0.110	0.081	0.061
r=4_h=0.4	0.304	0.178	0.111	0.081	0.061
r=4_h=0.5	0.306	0.177	0.110	0.081	0.061
r=4_h=0.6	0.302	0.179	0.112	0.081	0.061
r=4_h=0.7	0.308	0.190	0.116	0.083	0.061
r=4_h=0.8	0.257	0.214	0.145	0.103	0.068
r=8_h=0.2	0.303	0.176	0.111	0.080	0.061
r=8_h=0.3	0.304	0.175	0.111	0.081	0.062
r=8_h=0.4	0.306	0.176	0.112	0.082	0.061
r=8_h=0.5	0.303	0.181	0.112	0.082	0.062
r=8_h=0.6	0.282	0.181	0.113	0.082	0.062
r=8_h=0.7	0.301	0.208	0.125	0.086	0.065
r=8_h=0.8	0.248	0.163	0.162	0.111	0.079
r=10_h=0.2	0.303	0.175	0.109	0.080	0.061
r=10_h=0.3	0.304	0.174	0.110	0.079	0.061
r=10_h=0.4	0.306	0.173	0.111	0.080	0.062
r=10_h=0.5	0.303	0.178	0.114	0.082	0.062
r=10_h=0.6	0.276	0.180	0.116	0.082	0.062
r=10_h=0.7	0.301	0.202	0.131	0.092	0.067
r=10_h=0.8	0.247	0.157	0.140	0.119	0.082
r=12_h=0.2	0.304	0.175	0.110	0.079	0.062
r=12_h=0.3	0.305	0.173	0.109	0.079	0.062
r=12_h=0.4	0.307	0.174	0.112	0.082	0.063
r=12_h=0.5	0.298	0.178	0.117	0.083	0.063
r=12_h=0.6	0.272	0.183	0.118	0.084	0.063
r=12_h=0.7	0.289	0.207	0.138	0.091	0.069
r=12_h=0.8	0.243	0.159	0.121	0.130	0.083
r=14_h=0.2	0.304	0.175	0.108	0.080	0.062
r=14_h=0.3	0.305	0.173	0.109	0.080	0.061
r=14_h=0.4	0.307	0.173	0.110	0.082	0.062
r=14_h=0.5	0.292	0.178	0.118	0.083	0.062
r=14_h=0.6	0.264	0.182	0.120	0.084	0.062
r=14_h=0.7	0.286	0.202	0.141	0.097	0.072
r=14_h=0.8	0.244	0.159	0.117	0.118	0.093
