# Supplementary material for: Integrated Module and Gene-Specific Regulatory Inference Implicates Upstream Signaling Networks
Source: PLoS Comput Biol. 2013 Oct 17;9(10):e1003252. doi: 10.1371/journal.pcbi.1003252 (PMC3798279; doi:10.1371/journal.pcbi.1003252)
Supplement: Text S1 — Pseudo code and additional details of the MERLIN algorithm. (PDF) [file pcbi.1003252.s010.pdf]

Pseudo code and additional details of the MERLIN algorithm

As described in the text the MERLIN algorithm is an iterative algorithm that infers module-constrained per-gene regulatory networks. Here we describe the pseudo code for the MERLIN algorithm. There are two phases in the network inference: learning the regulators per gene (**Steps 7-13**) given the current module membership, and updating the module membership given the current regulator network (**Steps 15-18**). The algorithm starts with an empty regulator set $\mathbf{R}_i$ for each gene X_i. During the regulator identification steps (**Step 6-11**), it updates the $\mathbf{R}_i$ by identifying the next best regulator that improves the score of a gene X_i. It repeats this procedure for each target gene either until there is no more score improvement for X_i or a fixed number of steps have been executed. While it is adding regulators to a gene, it also updates the regulator-module relationship, which influences which regulators get selected for subsequent genes.

Once the regulator sets of all variables have been examined, we update the module memberships (**Steps 13-16**). This is done efficiently by making use of a min-heap data structure. We also do not merge any nodes that have a greater than the specified threshold of clustering. When we merge two nodes, k and l in the hierarchy we use average linkage to define the distance of the new node, m from all other nodes, n (**Step 15-16**). This step defines our modules. Next using these modules we update the regulators associated with each gene to see if adding more regulators helps improve the score associated with a gene.

Algorithm 1 Learning in MERLIN

1: **Input:**
   Initial module assignment for each gene, $\mathbf{M}_{init}$
   Dataset $\mathcal{D}$
   Candidate regulators $\mathcal{R}$
   Sparsity: p, Module prior: r, Minimum similarity between two modules: h
2: **Output:**
   Inferred module for each gene, $\mathbf{M}_{final}$
   Regulatory network, specifying the set of regulators, $\mathbf{R}$) per gene as well as their parameters, θ_i
3: $\mathbf{M}_{curr} = \mathbf{M}_{init}$
4: $\mathbf{R}_i = \emptyset, \forall i$ /*Initialize regulators for each gene*/
5: **while** not converged **do**
6:   */*Update regulators* $\mathbf{R}_i\ \forall X_i$ *given* $\mathbf{M}_{curr}$.*/
7:   **for** $X_i \in \mathbf{X}$ **do**
8:     **repeat**
9:       $X_k = \arg\max_{X_j \in \mathcal{R} \setminus \mathbf{R}_i} S(X_i; \mathbf{R}_i \cup X_j) - S(X_i; \mathbf{R}_i)$
10:       $\mathbf{R}_i = \mathbf{R}_i \cup \{X_k\}$.
11:       Add X_k to X_i's module, M_i.
12:     **until** A fixed number of iterations or until adding regulators does not improve X_i's score
13:   **end for**
14:   */*Hierarchically cluster genes using co-expression and co-regulator for pairs of genes to obtain new* $\mathbf{M}_c urr$**/.*
15:   **while** There exists a node pair k and l such that $dist(k, l) \leq h$ **do**
16:     Merge k, l into new node m.
17:     Compute distance $dist(m, n)$ for all nodes other than k and l and insert pair into min heap.
18:   **end while**
19: **end while**
20: $\mathbf{M}_{final} = \mathbf{M}_{curr}$

Parameter estimation in MERLIN

To compute the score $S(X_i, \mathbf{R}_i)$ for each gene, X_i and its regulators $\mathbf{R}_i$, we assume that X_i and $\mathbf{R}_i$ are distributed according to a $|\mathbf{R}_i| + 1$-dimensional multi-variate Gaussian, with mean $\mathbf{m}_i$, a $|\mathbf{R}_i| + 1$-dimensional mean vector, and a $|\mathbf{R}_i| + 1 \times |\mathbf{R}_i| + 1$-dimensional co-variance matrix $\mathbf{\Sigma}_i$. To estimate the conditional probability distributions of a gene's expression level given its regulators' expression level in sample d we estimate a conditional mean $\mu_{i|\mathbf{R}_i}$ and conditional variance $\Sigma_{i|\mathbf{R}_i}$ as follows:

$$\mu_{i|\mathbf{R}_i} = \mu_i + \mathbf{\Sigma}_i(i, -i)inv(\mathbf{\Sigma}_i(-i, -i))(\mathbf{x}^d_{\mathbf{R}_i} - \mathbf{m}_{-i})^{\mathsf{T}}.$$

$$\sigma_{i|\mathbf{R}_i} = \sigma_{ii} - \mathbf{\Sigma}_i(i, -i)inv(\mathbf{\Sigma}_i(-i, -i))\mathbf{\Sigma}^{\mathsf{T}}_i(i, -i).$$

Here μ_i is the mean expression level of gene i, σ_{ii} is the variance of X_i. $\mathbf{m}_{-i}$ is the mean of all elements in $\mathbf{R}_i$, and $\mathbf{x}^d_{\mathbf{R}_i}$ is the assignment to all elements of $\mathbf{R}_i$ in the dth sample. $\mathbf{\Sigma}_i(-i, -i)$ is the original $\mathbf{\Sigma}_i$ after dropping the row and column corresponding to X_i. $\mathbf{\Sigma}_i(i, -i)$ is the row in $\mathbf{\Sigma}_i(-i, -i)$ corresponding to X_i.
